# Delimiting species boundaries in *Hosta* section *Capitatae* (Asparagaceae) using MIG-seq and morphological analyses: taxonomic revision with new taxa from Korea and Japan

**DOI:** 10.3389/fpls.2025.1668561

**Published:** 2026-01-21

**Authors:** Ami Oh, Ji Young Yang, Won Seok Lee, Takashi Shiga, Seiko Fujii, Shota Sakaguchi, Shukherdorj Baasanmunkh, Seung-Chul Kim, Hyeok Jae Choi

**Affiliations:** 1 Division of Research Planning and General Affairs, Korea National Arboretum, Pocheon, Republic of Korea; 2 Global Institute for Advanced Nanoscience & Technology (GIANT), Changwon National University, Changwon, Republic of Korea; 3 Department of Biological Sciences, Sungkyunkwan University, Suwon, Republic of Korea; 4 Department of Biology and Chemistry, Changwon National University, Changwon, Republic of Korea; 5 Faculty of Education, Niigata University, Niigata, Japan; 6 Plant Research Division, The Kochi Prefectural Makino Botanical Garden, Kochi, Japan; 7 Graduate School of Human and Environmental Studies, Kyoto University, Kyoto, Japan

**Keywords:** *Capitatae*, *Hosta nakaiana* var. *wandoensis*, *H. pseudonakaiana*, MIG-seq, morphology, new species, population structure

## Abstract

**Introduction:**

*Hosta capitata*, which has recently been placed in the monotypic section *Capitatae*, and *H. nakaiana*, were originally described in Iya Valley, Japan and Mt. Baegun, Korea, respectively, and have been considered the same from a morphological perspective. However, considering the significant genetic distance between these groups, the identity of *H. nakaiana* deserves further investigations. Recently, the populations of *H. capitata* from Kochi, Japan, and Wando Island, Korea have been distinguished from the other groups by their distinctive morphological traits. On the basis of these observations, the present study aimed to provide a complete taxonomic revision of the section *Capitatae* in Korea and Japan based on extensive morphological observations and multiplexed inter-simple sequence repeats genotyping by sequencing (MIG-seq) analysis.

**Methods:**

Samples of the section *Capitatae* were collected from Japan and Korea. Comprehensive morphological observation of the section *Capitatae* was performed using both quantitative and qualitative characteristics, and Principal Component Analysis (PCA) was conducted with the quantitative characters. For the molecular analysis of the section *Capitatae*, MIG-seq library was constructed and SNPs were identified. A phylogenetic tree was inferred using the maximum likelihood (ML) method. The genetic structure of the section *Capitatae* was determined by performing the STRUCTURE analysis and generating the Principal Coordinate Analysis (PCoA) plot.

**Results:**

The quantitative characteristics exhibited clear separation between the Kochi lineage and the other groups of the section *Capitatae*, and the qualitative characteristics showed distinct division between the Wando population and the other groups. The PCA results clearly identified two distinct groups, the Kochi lineage and the others. In the phylogenetic tree, the monophyly of the section *Capitatae* was strongly supported, and the section *Capitatae* consisted of three distinct clusters, *H. capitata*, *H. nakaiana* and the Kochi lineage. The Kochi lineage was supported as monophyletic (96% BS), and the Wando population was embedded within *H. nakaiana* cluster. Both the STRUCTURE analysis and the PCoA identified clear genetic differentiation among *H. capitata*, *H. nakaiana* and the Kochi lineage.

**Discussion:**

Our findings identified three species and two varieties in the section *Capitatae*. In particular, we described two new taxa, *H. pseudonakaiana* sp. nov. and *H. nakaiana* var. *wandoensis* var. nov., from Japan (Shikoku) and Korea (southern Jeollanam-do), respectively. Our study provides the most comprehensive framework for the classification of the section *Capitatae*, ultimately advancing the taxonomy of the genus *Hosta*.

## 1 Introduction

The genus *Hosta* Tratt. is widely used in gardening because of its ground-covering, glossy foliage, leaf patterns, and showy purple-to-white flowers (Jones, 1989; Chung, 1990). It is characterized by a leaf base that is abruptly tapered into the petiole, six stamens, united tepals, and purple-to-white flowers (Chen and Boufford, 2000). With approximately 22–45 species, *Hosta* is naturally distributed in Korea, Japan, China, and the Russian Far East, mainly on rocky substrates, forest edges, or grasslands in alpine regions (Jones, 1989; Chen and Boufford, 2000; Chung, 2007; Yahara et al., 2023). *Hosta* was traditionally classified under Liliaceae (Maekawa, 1940; Fujita, 1976; Chung, 1990; Chung et al., 1991; Schmid, 1991) or Agavaceae (Moran, 1949; Takhtajan, 1980), but it has recently been placed in the subfamily Agavoideae in Asparagaceae (APG IV, 2016; Jo and Kim, 2017; Lee et al., 2018). The genera *Hemerocallis*, *Agave*, *Hesperocallis*, *Leucocrimum*, *Yucca*, *Manfreda*, and *Camassia* are the most closely related to *Hosta* (Chung and Jones, 1989; Liu et al., 2011).

The taxonomy within the genus *Hosta* is highly complex and confusing due to its natural and artificial hybridization, abundant cultivars, and extensively variable morphology (Lee et al., 2019; Yang et al., 2021; Yoo et al., 2021). Specifically, hybridization and introgression which are common in this genus, have complicated species delimitation of *Hosta* (Yang et al., 2021; Yoo et al., 2021). Many previous studies have revised the infrageneric classification of *Hosta*, reporting diverse taxonomic systems (Maekawa, 1940; Fujita, 1976; Schmid, 1991; Zonneveld and Van Iren, 2001). In general, three subgenera, *Hosta*, *Bryocles*, and *Giboshi*, have been recognized in *Hosta* (Schmid, 1991; Zonneveld and Van Iren, 2001; Chung, 2007; Yang et al., 2021) while different numbers of sections were identified within the genus (Schmid, 1991; Zonneveld and Van Iren, 2001). One of the most recent and frequently cited studies by Schmid (1991) reported ten sections in *Hosta*. Among these, *Hosta* sect. *Lamellatae* (F.Maek.) F.Maek. is morphologically characterized by an erect and ridged scape (Maekawa, 1938). The three species in this section, *Hosta capitata* (Koidz.) Nakai, *H. minor* (Baker) Nakai, and *H. venusta* F. Maek. are found in Korea and Japan (Maekawa, 1938; Chung, 2007; Tamura and Fujita, 2016). However, *H. capitata* has recently been placed in its monotypic sect. *Capitatae* (F.Maek.) J.C.Yang, due to its unique morphology, which is a compact spike-like raceme (Lee et al., 2018). Notably, in the genus *Hosta*, morphological characteristics such as the presence of rhizomes, stamen connection, shape of leaf, scape, inflorescence, and flower, have historically been employed in taxonomic studies and have supported a stable classification (Maekawa, 1938; Tamura and Fujita, 2016).

*Hosta capitata* and *H. nakaiana* F.Maek. were originally described in the Iya Valley, Shikoku, Japan (Nakai, 1930a) and Mt. Baegun, Jeollanam-do, Korea (Maekawa, 1935), respectively. However, from a morphological perspective, both species are considered the same, and thus, *H. nakaiana* is regarded as a synonym of *H. capitata* (Fujita, 1976; Tamura and Fujita, 2016; Jo and Kim, 2017). Therefore, *H*. *capitata* (=*H*. *nakaiana*) shows a disjunct distribution, occurring in the main mountain range (central and southern parts) of Korea and the western parts of Japan (Fujita, 1976; Chung et al., 1991).

In a chloroplast whole genome-based phylogenetic study by Yang et al. (2021), species belonging to the sect. *Lamellatae* s.l., including *H. minor*, *H. venusta*, and *H. capitata* (*=H. nakaiana*), did not form a single clade, and the monotypic sect. *Capitatae* was clearly established as a distinct evolutionary lineage. In terms of the pairwise sequence distance (K2P), the average distance between the Korean and Japanese populations of *H. capitata* (=*H. nakaiana*) was 0.00020 ± 0.00003. For comparison, the K2P between sister species in the genus *Hosta*, *H. jonesii* M.G.Chung and *H. tsushimensis* N.Fujita, and between *H. ventricosa* (Salisb.) Stearn and *H. sieboldiana* Engl. were 0.00026 and 0.00008, respectively. Additionally, the distance between *H. minor* and *H. venusta*, two species endemic to Korea, was 0.00003 ± 0.00002 (Yang et al., 2021). Consequently, the taxonomic identity of *H. nakaiana* remains controversial (Schmid, 1991; Sauve et al., 2005).

Recently, notable morphological variability has been recognized within *H. capitata*, where the population from Kochi, Japan, exhibits a clear morphological distinction from the other groups (personal observation). Specifically, the Kochi population is distinguished from other groups by narrower leaf and perianth lobes, and fewer flowers per inflorescence. This observation warrants further research to determine whether this population represents a distinct lineage. Meanwhile, during a field trip to Wando Island, Korea, we observed a population of *H. capitata* with a distinctively looser inflorescence, which deserves an in-depth investigation.

In this study, we combined morphological data with multiplexed inter-simple sequence repeats genotyping by sequencing (MIG-seq) (Suyama and Matsuki, 2015), a method well-suited for reconstructing phylogenetic relationships among closely related species, and population genetic analyses (Binh et al., 2018; Strijk et al., 2020; Zhang et al., 2020; Yahara et al., 2021; Kusuma et al., 2022; Tamaki et al., 2023; Yahara et al., 2023). This integrative approach was applied to revise the taxonomy of *Hosta* sect. *Capitatae* in Korea and Japan.

## 2 Materials and methods

### 2.1 Taxon Sampling

Samples of *H. capitata* (=*H. nakaiana*), including two putative new taxa, were collected from Japan and Korea during extensive field surveys between 2013 and 2022 (**Table 1**). Samples from Japan were collected from Mt. Rokko in Hyogo, Iya in Tokushima, and Kochi. Samples from Korea were collected from eight populations: Bonghwa, Mt. Baegun, Mt. Daedeok, Mt. Gamak, Mt. Worak, Mt. Songni, Mt. Irwol, and Wando. For the phylogenetic analysis, *H. minor*, *H. yingeri*, *H. jonesii*, and *H. venusta* were collected from their habitats in Korea (**Table 1**). Voucher specimens were deposited in the herbarium of Changwon National University, Republic of Korea (CWNU).

**Table 1:** List of *Hosta* samples used for phylogenetic analysis.

Scientific name	Voucher ID	Locality	Latitude and longitude
*H. capitata*	*T.Kurazono 160904-Minamirokko-001*	Mt. Rokko, Hyogo pref., Japan	Available on request
*H. capitata*	*190707-Rokko(Gobe)-001*	Mt. Rokko, Hyogo pref., Japan	Available on request
*H. capitata*	*T.Shiga 9261-9264*	Iya elementary school, Tokushima pref., Japan	Available on request
*H. capitata*	*161008-Iya-001*	Iya valley, Tokushima pref., Japan	Available on request
*H. jonesii*	*HJ-JD01*	Jeopdo Isl., Jeollanam-do, Korea	N34.37624 E126.29005
*H. jonesii*	*HJ-NH01*	Namhae natural forest, Gyeongsangnam-do, Korea	N34.75220 E128.01727
*H. jonesii*	*HJ-GS01*	Mt. Geumsan, Gyeongsangnam-do, Korea	N34.75228 E127.98214
*H. minor*	*HM-BM01*	Mt. Bulmosan, Gyeongsangnam-do, Korea	N35.18017 E 128.72355
*H. minor*	*HM-CR01*	Mt. Cheongnyangsan, Gyeongsangbuk-do, Korea	N36.78724 E128.89820
*H. minor*	*HM-HW01*	Mt. Haewolbong, Gyeongsangbuk-do, Korea	–
*H. minor*	*HM-JB01*	Mt. Jukbyeonsan, Gangwon-do, Korea	N38.30397 E128.46625
*H. minor*	*HM-HU01*	Mt. Hanusan, Gyeongsangnam-do, Korea	–
*H. minor*	*HM-SB01*	Mt. Sinbulsan, Gyeongsangnam-do, Korea	–
*H. minor*	*HM-GS01*	Gasa forest, Jeollanam-do, Korea	N34.37150 E126.92563
*H. nakaiana*	*HN-BH01*	Bonghwa, Gyeongsangbuk-do, Korea	–
*H. nakaiana*	*150806-Gwangyangsi(Baegunsan)-046*	Mt. Baegun, Jeollanam-do, Korea	N35.10241 E127.65130
*H. nakaiana*	*160729-Taebaeksi(Daedeoksan)-001*	Mt. Daedeok, Gangwon-do, Korea	N37.23967 E128.91963
*H. nakaiana*	HN-GA01	Mt. Gamak, Gyeongsangnam-do, Korea	–
*H. nakaiana*	HN-WR01	Mt. Worak, Chungcheongbuk-do, Korea	–
*H. nakaiana*	HN-IW01	Mt. Irwol, Gyeongsangbuk-do, Korea	–
*H. nakaiana*	*Sangjusi (Songnisan)-060621-112*	Mt. Songni, Chungcheongbuk-do, Korea	–
Wando population	*Wando-210623-001*	Wando, Jeollanam-do, Korea	N34.34675 E126.69531
Kochi lineage	*190706-Chojako(Kochi)-001*	Chojako, Kochi pref., Japan	Available on request
Kochi lineage	*T.Shiga 11296*	Chojahei, Kochi pref., Japan	Available on request
Kochi lineage	*M.J.Choi & T.Shiga 190706-001*	Niyodogawa, Kochi pref., Japan	Available on request
Kochi lineage	*HP-NG01*	Nagasawa, Kochi pref., Japan	Available on request
*H. venusta*	*HV-HL01*	Mt. Hallasan, Jeju-do, Korea	N33.35938 E126.46326
*H. venusta*	*HV-MY01*	Mt. Muryeongarioreum, Jeju-do, Korea	N33.36845 E126.69331
*H. venusta*	*HV-SA01*	Suakgyo Brdg., Jeju-do, Korea	N33.33699 E126.61111
*H. yingeri*	*HY-CL01*	Mt. Chillaksan, Jeollanam-do, Korea	N34.68240 E125.42640
*H. yingeri*	*HY-HD01*	Hongdo Isl., Jeollanam-do, Korea	–

Georeference data for Japanese localities are omitted to prevent unauthorized collection.

### 2.2 Morphological analysis

Morphological analyses were conducted using living plants from the habitats and herbarium specimens from the following herbaria: CWNU, KH, KYO, MBK, OSA, and TI (abbreviations are according to Thiers (2023)). The voucher specimens used for the measurement of morphological characteristics are marked with an asterisk (*) in the list of additional specimens examined. Photographs of the type specimens were also used for the comparative observation of key characteristics within the section. Some plants collected from these habitats were transplanted into an experimental field at Changwon National University to observe their growth patterns.

**Figure 1:**
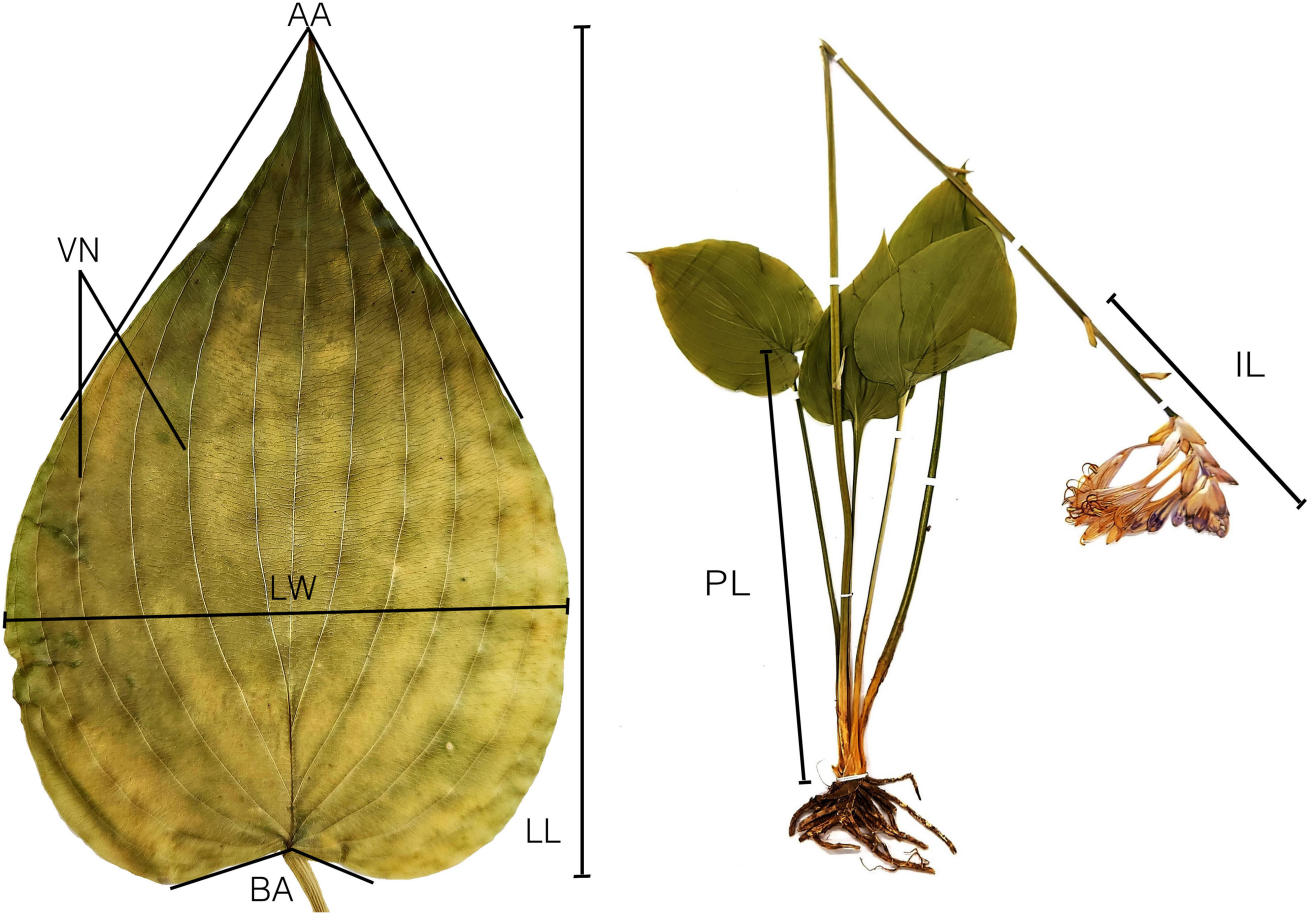
Diagram showing the morphological characters of *Hosta* sect. *Capitatae* measured in this study. LL, blade length (mm); LW, blade width (mm); AA, apex angle (°); BA, base angle (°); VN, lateral vein number (no.); LR, ratio of blade length to width (L:W); PL, petiole length (mm); IL, inflorescence length (mm); FN, flower number (no.).

**Figure 2:**
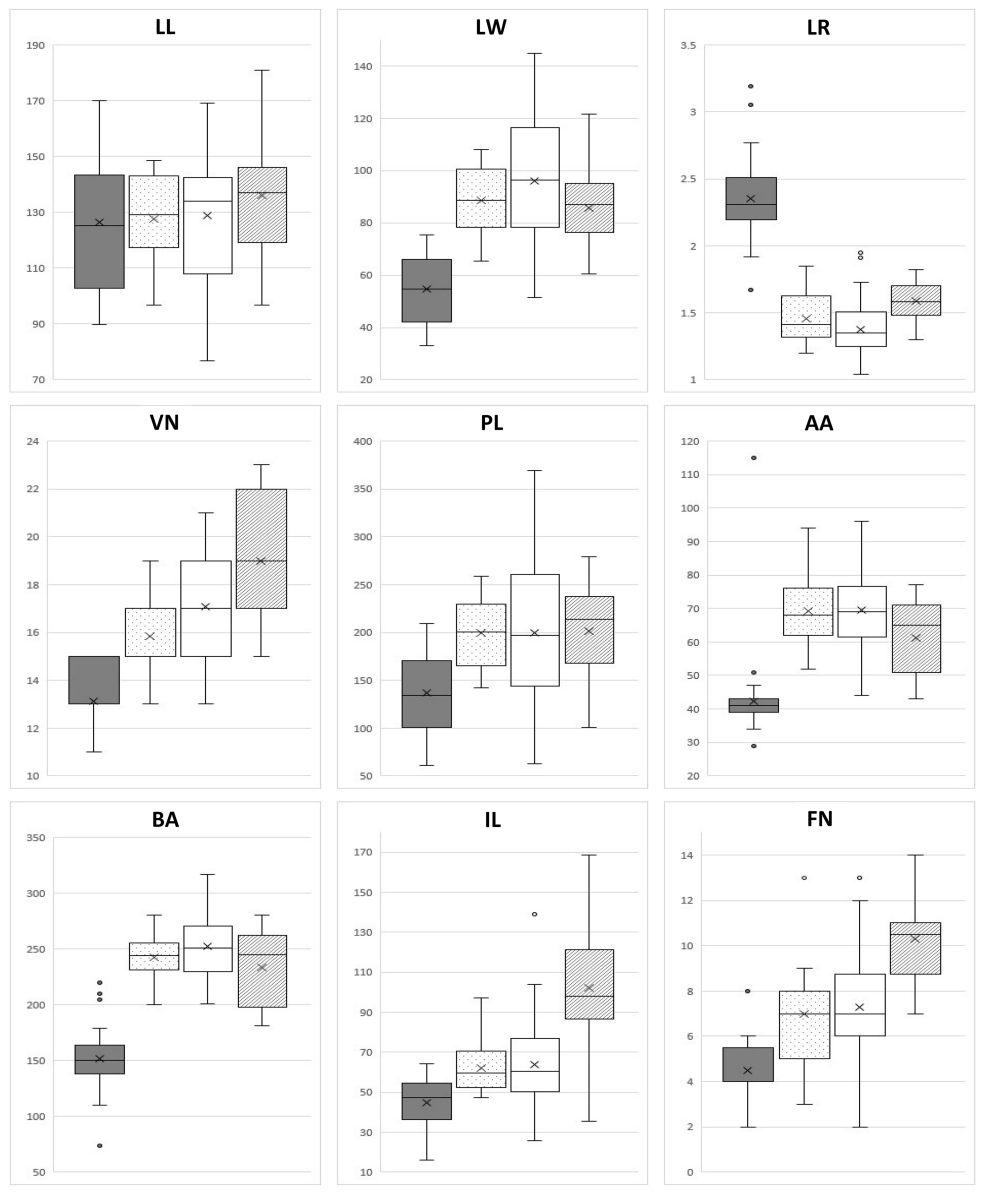
Quantitative data of morphological characteristics of four *Hosta* taxa. Boxplots show the median, 25^th^ and 75^th^ percentiles (box), 10^th^ and 90^th^ percentiles (whiskers), and outliers (closed circle). From left to right: Kochi lineage, *H. capitata*, *H. nakaiana*, Wando population. LL, blade length (mm); LW, blade width (mm); AA, apex angle (°); BA, base angle (°); VN, lateral vein number (no.); LR, ratio of blade length to width (L:W); PL, petiole length (mm); IL, inflorescence length (mm); FN, flower number (no.). Units of the y-axes are mm for LL, LW, PL, IL, degree (°) for AA, BA, and count (no.) for VN, FN.

Nine quantitative characteristics, including leaf blade length (LL), leaf blade width (LW), leaf apex angle (AA), leaf base angle (BA), leaf lateral vein number (VN), ratio of leaf length to width (LR), petiole length (PL), inflorescence length (IL), and flower number (FN), were measured and statistically analyzed (**Table 2**, **Figures 1**, **2**). Length and width were measured in 0.01 mm units using electronic Vernier calipers (Mitutoyo Corp. CD-15APX), and angles in 1° units using a protractor. The measured traits were organized into tables in CSV files and used for principal component analysis (PCA). The qualitative characteristics, such as the shape of the leaf, inflorescence, and perianth, were also investigated (**Table 3**).

**Table 2:** Comparison of quantitative characters of *Hosta* sect. *Capitatae* in Korea and Japan.

Character	Measurement minimum (mean ± SD) and maximum			
	Kochi lineage	*H. capitata*	*H. nakaiana*	
			Other populations	Wando population
Leaf				
Blade length (LL, mm)	89.7–170 (126.49±24.14)	96.79–148.5 (127.69±15.86)	76.76–169.18 (128.72±21.82)	96.7–194.66 (136.16±24.21)
Blade width (LW, mm)	33.16–75.27 (54.66±12.70)	65.23–108.02 (88.69±13.11)	51.54–144.89 (96.04±23.77)	60.66–121.58 (85.83±14.07)
Apex angle (AA, °)	29–115 (42.41±11.92)	52–94 (69.26±10.26)	44–96 (69.42±11.15)	43–77 (61.24±10.66)
Base angle (BA, °)	74–220 (152.07±27.10)	200–280 (242.26±20.74)	201–317 (252.40±27.91)	181–280 (233.06±32.33)
Lateral vein number (VN, no.)	11–15 (13.14±1.37)	13–19 (15.84±1.76)	13–21 (17.07±2.31)	15–23 (19±2.83)
Ratio of blade length to width (LR, L:W)	1.67–3.19 (2.35±0.27)	1.20–1.85 (1.45±0.18)	1.04–1.95 (1.38±0.19)	1.30–1.82 (1.59±0.14)
Petiole length (PL, mm)	60.82–208.97 (137.25±41.31)	141.87–258.6 (199.90±33.68)	62.32–368.7 (199.91±72.17)	100.69–279.33 (201.44±47.68)
Flower				
Inflorescence length (IL, mm)	16.2–64.11 (44.95±13.21)	47.34–97.2 (62.00±13.18)	25.7–139.04 (63.67±20.41)	35.71–168.59 (102.10±29.99)
Flower number (FN, no.)	2–8 (4.48±1.38)	3–13 (7±2.25)	2–13 (7.29±2.34)	7–14 (10.29±1.83)

SD, Standard Deviation.

**Table 3:** Comparison of qualitative characters of *Hosta* sect. *Capitatae* in Korea and Japan.

Character	Kochi lineage	*H. capitata*	*H. nakaiana*	
			Other populations	Wando population
Leaf				
Shape	Narrowly ovate	Ovate to broadly ovate	Ovate to broadly ovate	Broadly ovate
Base	Rounded to subcordate	Subcordate to cordate	Subcordate to cordate	Subcordate to cordate
Apex	Acuminate to acute	Acute to obtuse	Acute to obtuse	Acute to obtuse
Inflorescence				
Shape	Compact spike-like raceme	Compact spike-like raceme	Compact spike-like raceme	Loose spike-like raceme
Flowering season	June to July	End of June to July	End of June to July	Early to end of June
Perianth				
Shape	Bell shape	Bell shape	Bell shape	Bell shape
Lobe	Lanceolate	Broadly ovate	Broadly ovate	Broadly ovate

**Figure 3:**
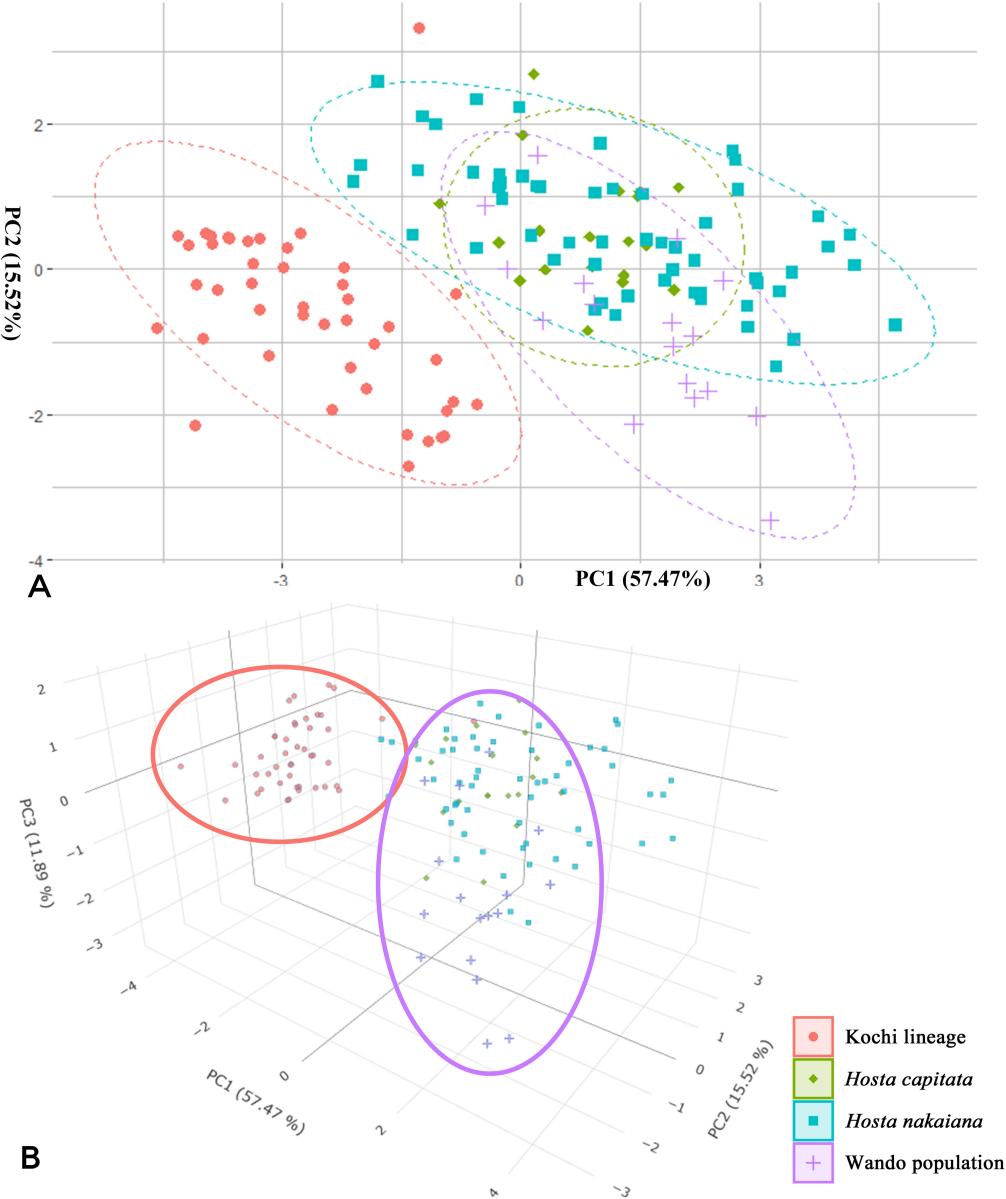
Cluster analysis based on quantitative morphological factors of four *Hosta* taxa. **(A)** Scatter plot of the first two principal components PC1-PC2. **(B)** Three-dimensional scatter plot. The red oval indicates the cluster of the Kochi lineage and the purple oval indicates the cluster of the Wando population.

PCA was performed for nine quantitative characters using FactoMineR v.1.04, stats v.4.1.3, and vegan packages v.2.0–10 in R v.4.1.3 (**Figure 3**, **Table 4**; Lê et al., 2008; Oksanen et al., 2013; R Core Team, 2022). The FactoMineR package was used to standardize numerical variables. The stats package was used to cluster the data of the nine quantitative factors. To assess the statistical reliability of the clusters, we performed a multivariate analysis of variance (MANOVA) using the vegan package (**Table 5**).

**Table 4:** Loading values of the three principal components using 9 quantitative characters of the *Hosta* sect. *Capitatae*.

Character	Component		
	PC1	PC2	PC3
LL	0.2196	-0.6251*	0.3663
PL	0.3645*	-0.2397	0.3145
LW	0.4151*	-0.0365	0.2281
VN	0.3763*	-0.0963	-0.0510
AA	0.3394	0.4379*	0.0162
BA	0.3616*	0.2869	0.0142
IL	0.2042	-0.2699	-0.6597*
FN	0.2663	-0.2375	-0.5256*
LR	-0.3827*	-0.3712	0.0169
Eigenvalue (%)	57.47	15.52	11.89
Cumulative of eigenvalue (%)	57.47	72.99	84.88

Components loaded highly for each character are denoted by asterisks (*).

Abbreviations of characteristics are the same as those in **Table 2**.

**Table 5:** Summary table of multivariate analysis of variance (MANOVA).

Source of variation	DF	Pillai	Approx. F	num DF	den DF	Pr(>F)
Lineage	2	1.4683	12.426	28	126	< 2.2e-16***
Residuals	75					

Significance Code: [0, 0.001] ‘***’.

### 2.3 DNA isolation, MIG-seq library construction, and genotyping

Total DNA was extracted from the silica gel-dried leaves using a DNeasy Plant Mini Kit (Qiagen, Carlsbad, CA, USA). The MIG-seq library for 192 samples of *H. capitata*, *H. nakaiana*, the Kochi lineage, *H. venusta*, *H. minor*, *H. yingeri* and *H. jonesii* was constructed using two-step amplification (Suyama and Matsuki, 2015). To amplify the target inter-simple sequence repeats (ISSR), we conducted the first PCR using ISSR primers developed by Suyama and Matsuki (2015). A second PCR was performed to add individual indices to each sample using barcoding primers designed by Suyama and Matsuki (2015). The amplicons were pooled and purified in selected sizes of 350–800 bp, and sequenced on an Illumina MiSeq platform (Illumina, San Diego, CA, USA) using the Illumina MiSeq Reagent Kit V3 (150 cycles, Illumina). The ‘DarkCycle’ option was used to skip the sequencing of SSR primer regions and anchors. Low-quality reads and extremely short reads containing index tags were removed from the next generation sequencing (NGS) data using FASTX Toolkit 1 (RRID: SCR_005534) and TagDust 1.12 (Lassmann et al., 2009). To maintain the quality of the dataset, 13 samples with extensive missing data were removed after screening using vcftools (Danecek et al., 2011). To identify SNPs in the processed reads, we used STACKS v.1.48 (Catchen et al., 2013) and generated data matrices in multiple formats (STURCTURE, Phylip, and variant call format (VCF)) for subsequent phylogenetic and population genetic analyses. Specifically, in STACKS, we applied sequential analyses that are ‘ustacks’, ‘cstacks’, and ‘sstacks’ with the following parameters: the minimum depth of coverage required to create a stack (m) = 3, maximum distance between stacks (M) = 2, and maximum mismatches between loci when building the catalog (n) = 2. In addition, we conducted ‘population’ analysis with the following parameters: at least 75% (–r 0.75) of the minimum percentage of individuals across populations, minimum two populations present to process a locus (–p 2), minimum minor allele frequency at a locus of 0.05 (–min-max 0.05), and maximum observed heterozygosity at a locus of 0.6 (–max-obs-het 0.6).

### 2.4 Phylogenetic and population structure analyses

A phylogenetic tree based on genome-wide 3,165 SNPs in the MIG-seq dataset for 176 *Hosta* samples was inferred using the maximum likelihood (ML) method implemented in IQ-TREE v.1.6.12 (Nguyen et al., 2015) with the best-fit model of “TVM+F+ASC+R2”. The bootstrap support value (BS) was calculated from 1,000 bootstrap replicates, using Ultrafast bootstrapping implemented in IQ-TREE. To determine the population structure of the section *Capitatae*, STRUCTURE v.2.3.4 (Pritchard et al., 2000) was used. The analysis was run for 147 samples of *H. capitata*, *H. nakaiana*, and the Kochi lineage, and the log-likelihood for each model was estimated using different numbers of populations (*K* = 2–9). The optimal *K* value was determined using the Delta *K* method described by Evanno et al. (2005) in Clumpak (Kopelman et al., 2015). Finally, to examine the genetic similarities and relationships among the individuals, Principal Coordinate Analysis (PCoA) was conducted by calculating the eigenvalues and eigenvectors using GenoDive v.3.06 (Meirmans, 2020). We used R v.4.1.3 (R Core Team, 2022) to construct the PCoA plot.

## 3 Results

### 3.1 Morphological characters

In our study, among the nine quantitative characteristics assessed, leaf blade width, ratio of leaf blade length to width, and angle of leaf apex and base exhibited clear separation between the Kochi lineage and the other groups of the section *Capitatae* (**Table 2**; **Figure 2**). Regarding qualitative characteristics, the shape of the inflorescence and flowering time showed distinct division between the Wando population and other groups (**Table 3**). PCA was performed using the nine quantitative trait data (**Figure 3**), with the first three principal components accounting for 84.88% of the total variance. PC1 accounted for 57.47% of the total variance, PC2 for 15.52%, and PC3 for 11.90%. When examining the contribution of leaf data to the principal components, PC1 showed substantial involvement, encompassing attributes such as leaf blade width, length-to-width ratio, base angle, petiole length, and vein number (**Table 4**). PC2 also exhibited contributions from the leaf data, specifically leaf blade length and apex angle (**Table 4**). In contrast, flower and inflorescence data, including inflorescence length and flower number per inflorescence, solely influenced PC3 (**Table 4**). The PCA results identified two groups: the Kochi lineage and others (**Figure 3**). Within the cluster other than the Kochi lineage, *H. capitata* showed high morphological similarity to *H. nakaiana* in Korea. Notably, the Wando population of *H. nakaiana* was slightly separated from the remaining taxa within the cluster. This group showed looser spike-like racemes and more flowers (**Table 3**). The morphological PCA results were supported by multivariate analysis of variance (MANOVA) (**Table 5**).

### 3.2 Phylogenetic tree reconstruction using MIG-seq

**Figure 4:**
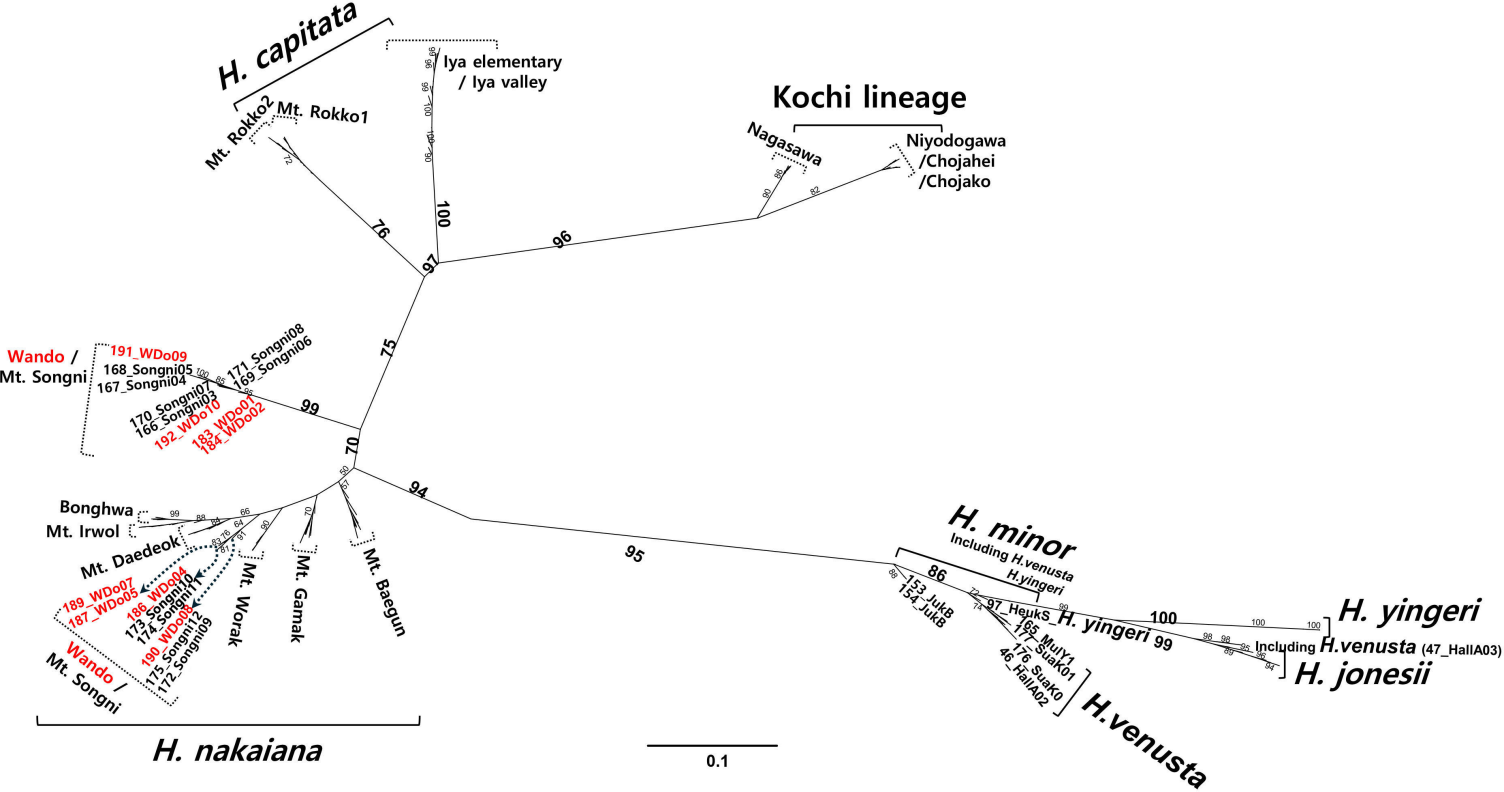
Unrooted Maximum likelihood (ML) tree generated using IQ-TREE based on the MIGseq-derived 3165 SNPs from 147 accessions of sect *Capitatae* (*H. capitata*, *H. nakaiana*, and Kochi lineage). Numbers on branches represent bootstrap support (BS) values of > 50%, based on 1000 replicates. Red letters indicate accessions collected from Wando, Korea.

In the process of MIG-seq data generation, where the 1^st^ PCR and the 2^nd^ PCR were conducted, PCR products were successfully obtained for all the 96 primer combinations in the 2^nd^ PCR. The number of loci recovered ranged from 1,617 to 15,759 (**Supplementary Table 1**). In total, 63,613,860 raw reads (144,483 ± 52,080 reads per sample) were obtained using MIG-seq. After quality control, 4,165,030 reads (21,806 ± 8,550 reads per sample) were used for further analyses. To infer the phylogenetic relationships among the 176 accessions of *Hosta* species, including *H. jonesii*, *H. minor*, *H. venusta*, *H. yingeri*, and the section *Capitatae* (*H. capitata*, *H. nakaiana*, and the Kochi lineage), ML analysis was performed (**Figure 4**). The unrooted ML tree strongly supported the monophyly of the section *Capitatae* (94% bootstrap value; BS). The section *Capitatae* consisted of three distinct clusters: the Kochi lineage, *H. capitata*, and *H. nakaiana*, which were genetically distant from the remaining species. The cluster of the Kochi lineage was recovered as monophyletic (96% BS) and was composed of two geographically distinct groups from the Nagasawa and Shikoku/Niyodo regions. *Hosta capitata*, which was most closely related to the Kochi lineage in the tree, was paraphyletic and consisted of two geographically differentiated groups from Mt. Rokko and the Iya region. Within *H. nakaiana*, which was recovered as paraphyletic and included eight populations from Korea, most populations showed monophyly, whereas other populations, such as those from Wando and Mt. Songni, did not. Notably, two clades composed of samples from both Wando and Mt. Songni were embedded in *H. nakaiana*, with bootstrap values of 91% and 99%. Among the other *Hosta* species, *H. minor*, *H. yingeri*, *H. venusta*, and *H. jonesii*, *H. jonesii* alone was identified as monophyletic (99% BS). *Hosta venusta*, a Korean endemic species, was embedded in both *H. yingeri* and *H. minor* clusters, whereas an individual of *H. yingeri* was nested within the *H. minor* cluster. The *H. yingeri* cluster was sister to the *H. jonesii* cluster, and the cluster that included both *H. yingeri* and *H. jonesii* appeared to be sister to the *H. minor*/*H. venusta* cluster.

### 3.3 Population structure analyses using MIG-seq

**Figure 5:**
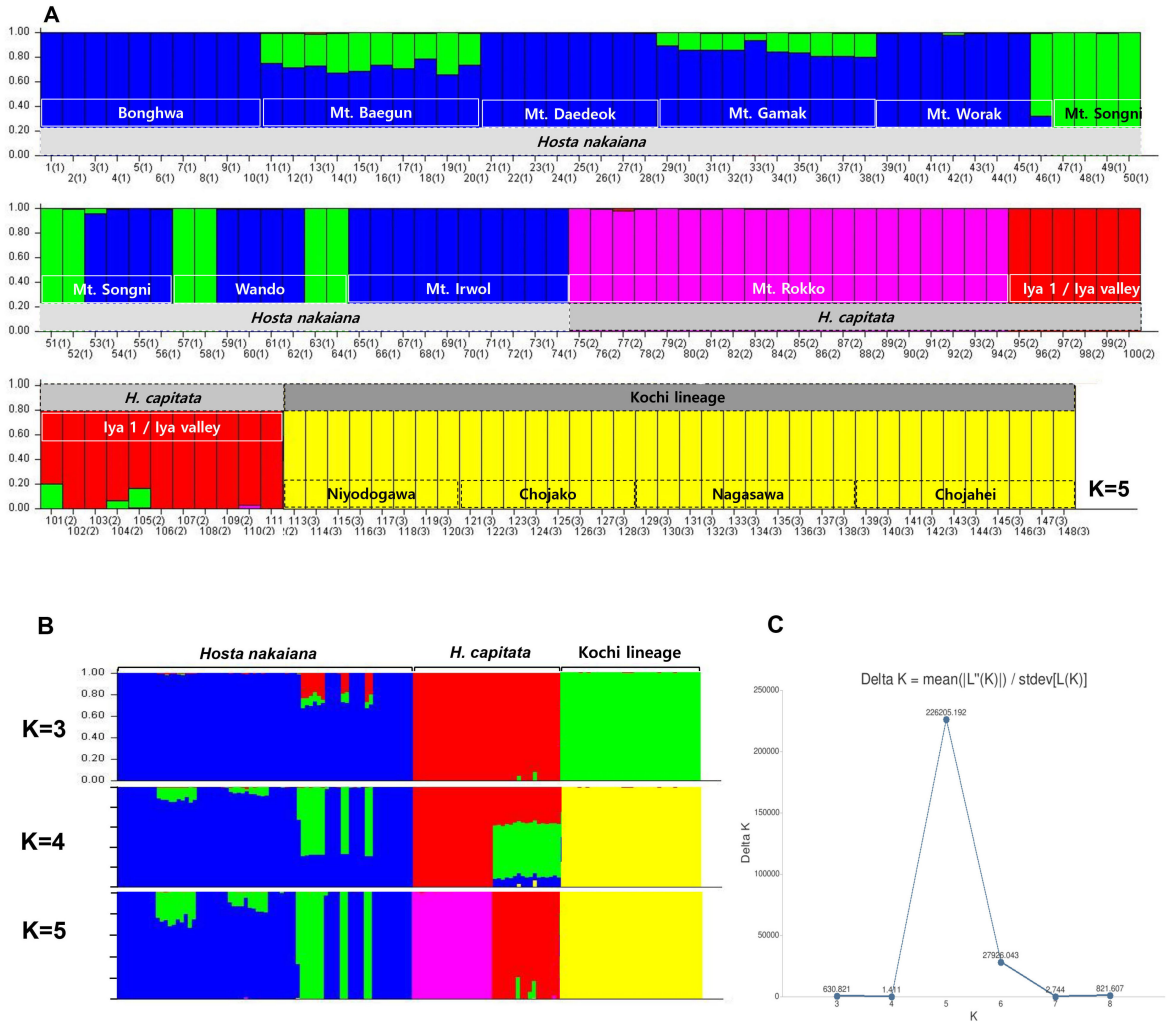
**(A)** Bar plot of STRUCTURE analysis for 147 accessions of *Hosta* sect. *Capitatae* (*H. capitata*, *H. nakaiana*, and Kochi lineage) at ΔK = 5. **(B)** Comparative bar plots showing population structure at K = 3, 4, and 5. **(C)** Delta K distribution graph used to identify the best-fit K value using the method of Evanno et al. (2005) in Clumpak.

The genetic structures of 147 accessions from the three lineages of the section *Capitatae*, the Kochi lineage, *H. capitata*, and *H. nakaiana*, were determined using genetic assignment analysis (**Figures 5A, B**). The optimal K, based on the ΔK method, was 5 (**Figures 5B, C**). When K = 5 (**Figure 5A**), the individuals of *H. nakaiana* were assigned to two clusters: cluster 1 is represented in blue and cluster 2 in green, although most of the individuals were assigned to cluster 1. Individuals from Bonghwa, Mt. Daedeok, and Mt. Irwol were assigned to cluster 1, whereas all individuals from Mt. Baegun and Mt. Gamak showed slight admixture signals. The Mt. Songni and Wando populations contained three and four individuals that were completely assigned to cluster 1, whereas six and four individuals from each population were classified as pure cluster 2. In the case of *H. capitata*, individuals from Mt. Rokko and the Iya region were separately assigned to two clusters, cluster 3 represented in pink and cluster 4 in red. All the individuals of the Kochi lineage were completely assigned to cluster 5 in yellow.

**Figure 6:**
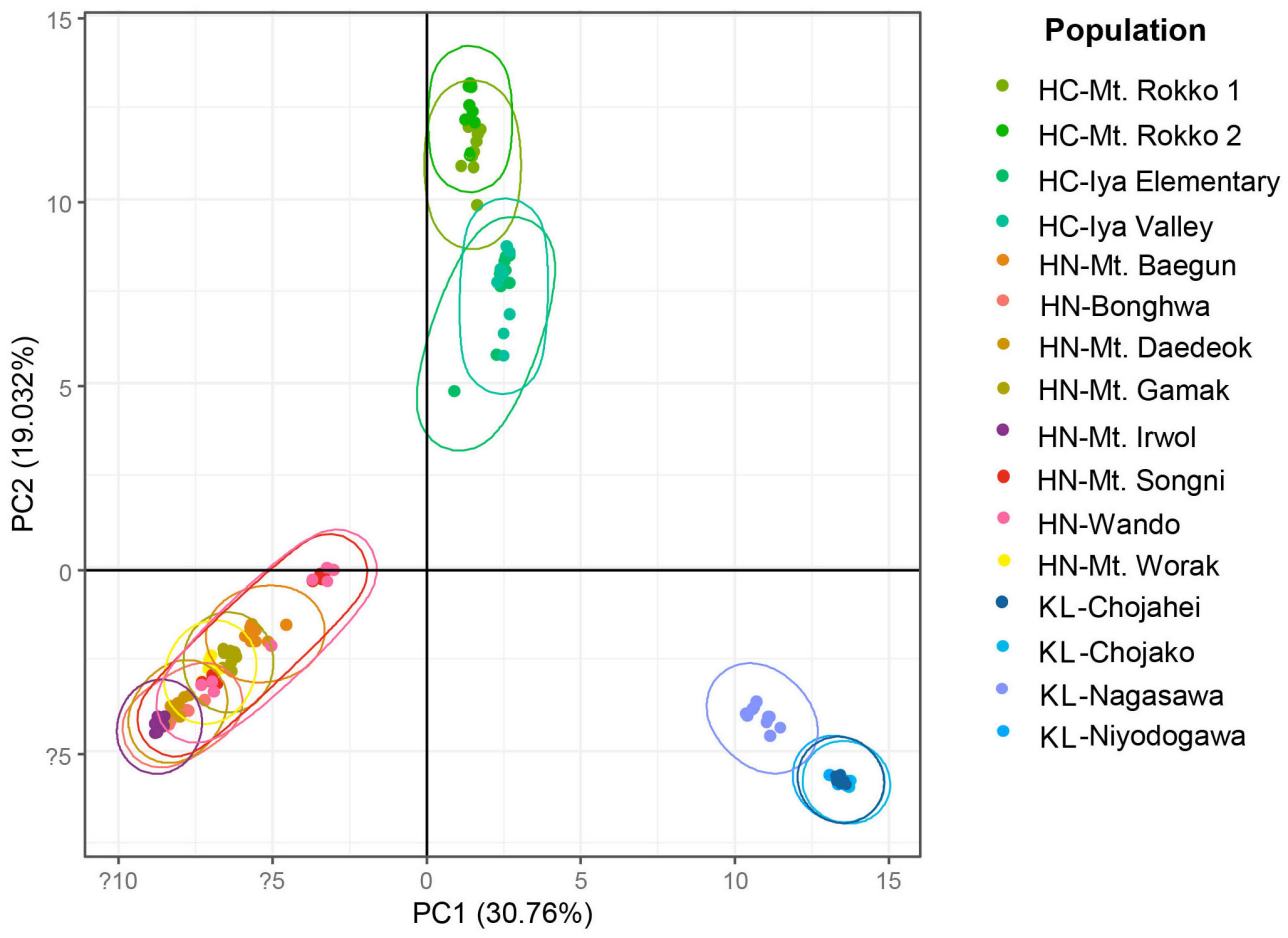
Principal coordinate analysis (PCoA) score plot for 147 accessions of sect *Capitatae* (*H. capitata* (HC), *H. nakaiana* (HN) and Kochi lineage (KL)).

The genetic structures of the three lineages of the section *Capitatae* assessed using PCoA (**Figure 6**) showed genetic differentiation between *H. capitata*, *H. nakaiana*, and the Kochi lineage. The PC1 axis distinguished all three groups, whereas *H. nakaiana* and the Kochi lineage were separated from *H. capitata* along the PC2 axis. In the plot, *H. nakaiana* comprised continuously distributed genetic clusters, with some individuals from Wando and Mt. Songni located separately. In *H. capitata*, two genetically and geographically differentiated clusters were observed. Furthermore, in the Kochi lineage, two distinct genetic clusters were identified: the Nagasawa population and others. All the populations of the Kochi lineage, except for the Nagasawa population, were completely clustered.

## 4 Discussion

### 4.1 Taxonomic revision of the section *Capitatae*

*Hosta*, popular in landscaping, medicine, and horticulture, has been long investigated taxonomically; however, species delimitation in this genus has been challenging (Li et al., 2012; Yang et al., 2021; Yoo et al., 2021). Notably, the monotypic section *Capitatae* has been only recently introduced into the genus (Lee et al., 2018), and its taxonomy, based on morphological or phylogenetic analyses, is largely lacking. In this study, the taxonomy of the section *Capitatae* was reassessed based on comprehensive morphological observations, phylogenetic inferences, and population structure analyses. We identified three species and two varieties in the section *Capitatae*. The Kochi lineage from Shikoku, Japan, was proposed as a new species and the Wando population from Korea as a new variety, contributing to the establishment of an advanced classification system for the section *Capitatae*.

Our findings indicate that the morphological characteristics analyzed in our study are of taxonomic utility in the section *Capitatae*. Specifically, the quantitative characteristics measured exhibited separation between the Kochi lineage in Shikoku, Japan, and the other groups inhabiting the southern part of Korea and Japan (**Table 2**, **Figure 2**). In addition, PCA results divided the section into two major groups: the Kochi lineage and remaining group (**Figure 3**). Phylogenetic and population structure analyses based on MIG-seq clarified and supported the morphology-based taxonomy of the section *Capitatae* (**Figures 4**, **5A**). In the phylogenetic tree, the Kochi lineage displayed monophyly, supporting its distinct entity as a new taxon (**Figure 4**). Notably, in the tree, *H. capitata* and the Kochi lineage were closely related; however, the degree of genetic differentiation was higher between these two groups than between *H. jonesii* and *H. yingeri*, two well-established *Hosta* species (**Figure 4**, Yang et al., 2021). This observation supports that the Kochi lineage should be considered a new species in the section *Capitatae*. In addition, the results of the STRUCTURE analysis, in which the Kochi lineage was assigned to a single distinct cluster, suggested that this lineage is genetically independent and should be treated as a novel species (**Figure 5A**). Finally, PCoA successfully supported the clear genetic separation of the Kochi lineage from the remaining taxa (**Figure 6**). Notably, the detection of a distinct Nagasawa population in the Kochi lineage confirmed the results of the phylogenetic analysis (**Figures 4**, **6**).

Although *H. nakaiana* was previously treated as a synonym of *H. capitata* (Tamura and Fujita, 2016; Jo and Kim, 2017) and our morphological analysis strongly supported the high similarity between them (**Tables 2**, **3**; **Figures 2**, **3**), our phylogenetic tree showed that *H. capitata* and *H. nakaiana* were genetically separate (**Figure 4**). STRUCTURE analysis revealed that these two groups had distinct genetic structures (**Figure 5A**). Furthermore, PCoA exhibited significant genetic separation between these taxa (**Figure 6**). A recent study by Yang et al. (2021) recognized that *H. capitata* populations in Japan and Korea are reciprocally monophyletic and genetically differentiated. Considering that the pairwise sequence distance between *H. capitata* in Japan and *H. nakaiana* in Korea is comparable to or larger than those between well-established *Hosta* species (Yang et al., 2021), it is highly likely that *H. nakaiana* is a different species from *H. capitata* and not a synonym. Notably, however, the sequence distances mentioned in our study, which are based on plastomes, should be interpreted with caution given that sequence divergence in plastomes of *Hosta* is quite low. Meanwhile, the identical morphology of these two groups indicates that *H. nakaiana* may be a cryptic species of *H. capitata* with divergent genetic and evolutionary traits.

The Wando population of *H. nakaiana* exhibits distinct morphological and phenological traits compared to the other groups, supporting its recognition as a novel taxon. In a previous phylogenetic analysis based on whole plastomes, the Wando population was genetically separated from the remaining *H. capitata* populations in Korea and Japan (**Supplementary Figure 1**). However, based on the results of the present study where the Wando population is deeply embedded in *H. nakaiana*, this group should be described as a new variety rather than a new species (**Figure 4**). Here, the Wando population is considered an incompletely distinct lineage of *H. nakaiana*. Meanwhile, phylogenetic analysis identified two clades containing samples from both Wando and Mt. Songni, indicating that these populations are genetically closely related (**Figure 4**). In addition, the STRUCTURE analysis revealed that the genetic compositions of the Mt. Songni and Wando populations share similarities (**Figure 5A**). Overall, the close relationship between the Wando and Mt. Songni populations in the phylogenetic tree, STRUCTURE plot, and PCoA plot suggests that the genetics, morphology, and evolution of these two populations should be investigated in parallel to better characterize this new variety from Wando.

In the previous studies on *Hosta*, the phylogenetic relationships among the species native to Korea, such as *H. minor*, *H. venusta*, *H. yingeri*, and *H. jonesii*, have been investigated in depth, and have shown both consistent and contrasting results (Lee et al., 2019; Yang et al., 2021; Yoo et al., 2021). At the same time, these species generally exhibited monophyly in these studies (Yang et al., 2021; Yoo et al., 2021), although Yoo et al. (2021) observed that the individuals of *H. venusta* were embedded within *H. minor* clade. In our phylogenetic analysis, regarding species genetically distant from the section *Capitatae* in the tree, *H. yingeri* cluster was sister to *H. jonesii* cluster, whereas the cluster comprising *H. yingeri* and *H. jonesii* was genetically close to *H. minor*/*H. venusta* group (**Figure 4**). Interestingly, the rough pattern of the genetic relationships aforementioned is consistent with the result of a previous phylogenomic analysis based on whole plastome sequences as well as that of another phylogenetic study that used the Hyb-Seq method, except that our analysis generally did not exhibit monophyly of the species (Yang et al., 2021; Yoo et al., 2021). However, in another phylogenetic analysis based on 246 nuclear genes, *H. yingeri*, the second most basal clade to the other *Hosta* species included, did not display sisterhood with *H. jonesii* (Yoo et al., 2021). Further phylogenetic analyses using more samples and various markers are needed to resolve this inconsistency and clarify the relationships between these *Hosta* species native to Korea.

Our study provides the most comprehensive framework for the classification of the section *Capitatae*, ultimately strengthening the taxonomy of the genus *Hosta*. This study also effectively complements Korean and Japanese flora by identifying the distributional patterns of newly reported taxa within the section *Capitatae*. Notably, in our study, the report of the new species and variety involves both morphological evaluation and molecular analysis, which greatly improved the quality and reliability of the report. Moreover, for the first time, our study used MIG-seq to successfully revise and update the taxonomy of the section *Capitatae*. Future studies employing genome-wide molecular data, extensive sampling, and state-of-the-art analysis tools may further elucidate the taxonomy of the *Hosta* sect. *Capitatae* based on the findings from our study.

### 4.2 Taxonomic treatment

A key to the species of *Hosta* sect. *Capitatae*

1. Leaves narrowly ovate, with leaf blade length:width ratio 1.67–3.19, perianth lobe lanceolate ————— *H. pseudonakaiana*

1. Leaves ovate to broadly ovate, with leaf blade length:width ratio 1.04–1.95, perianth lobe broadly ovate ————————— 2

2. Leaf blade length:width ratio 1.45 ± 0.18, 9.7–14.9 cm long, 6.5–10.8 cm wide, base truncate to subcordate ———— *H. capitata*

2. Leaf blade length:width ratio 1.38 ± 0.19, 7.7–16.9 cm long, 5.6–14.5 cm wide, base subcordate to cordate ——— *H. nakaiana*

**Figure 7:**
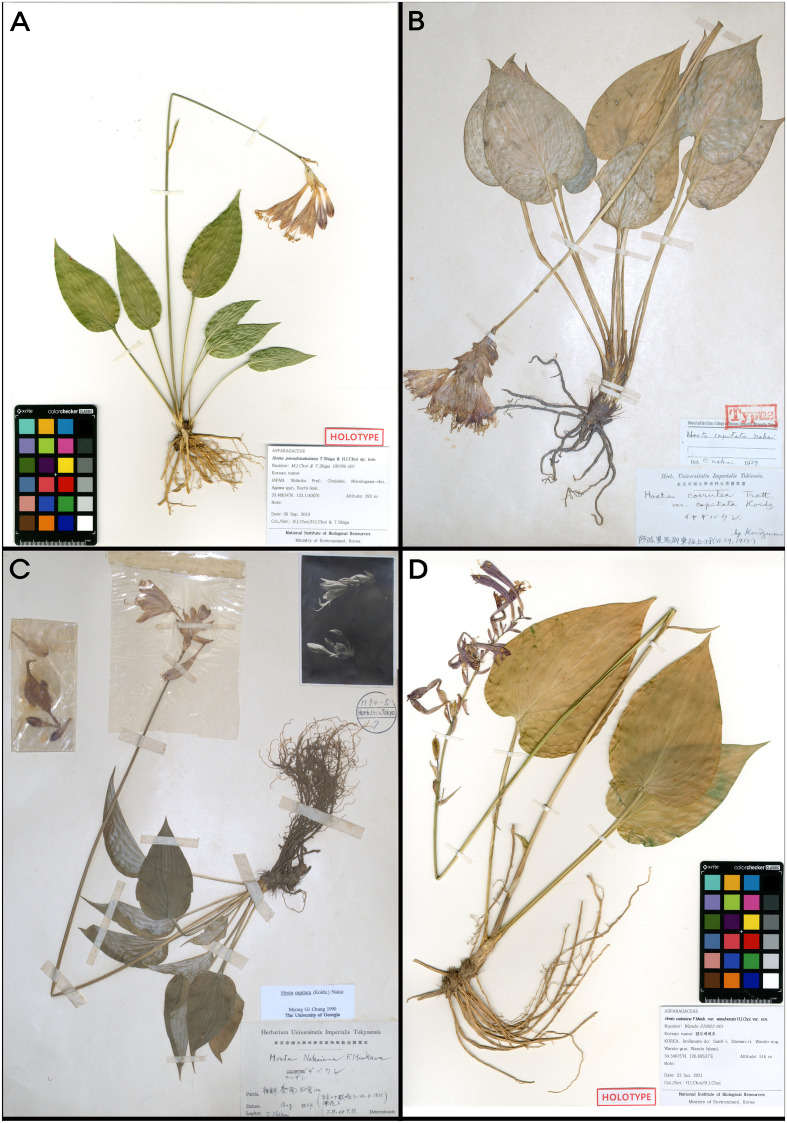
Type specimens of *Hosta* sect. *Capitatae*. **(A)***H. pseudonakaiana* (holotype KB); **(B)***H. capitata* (holotype TI); **(C)***H. nakaiana* var. nakaiana (holotype TI); **(D)***H. nakaiana* var. wandoensis (holotype HIBR).

**Figure 8:**
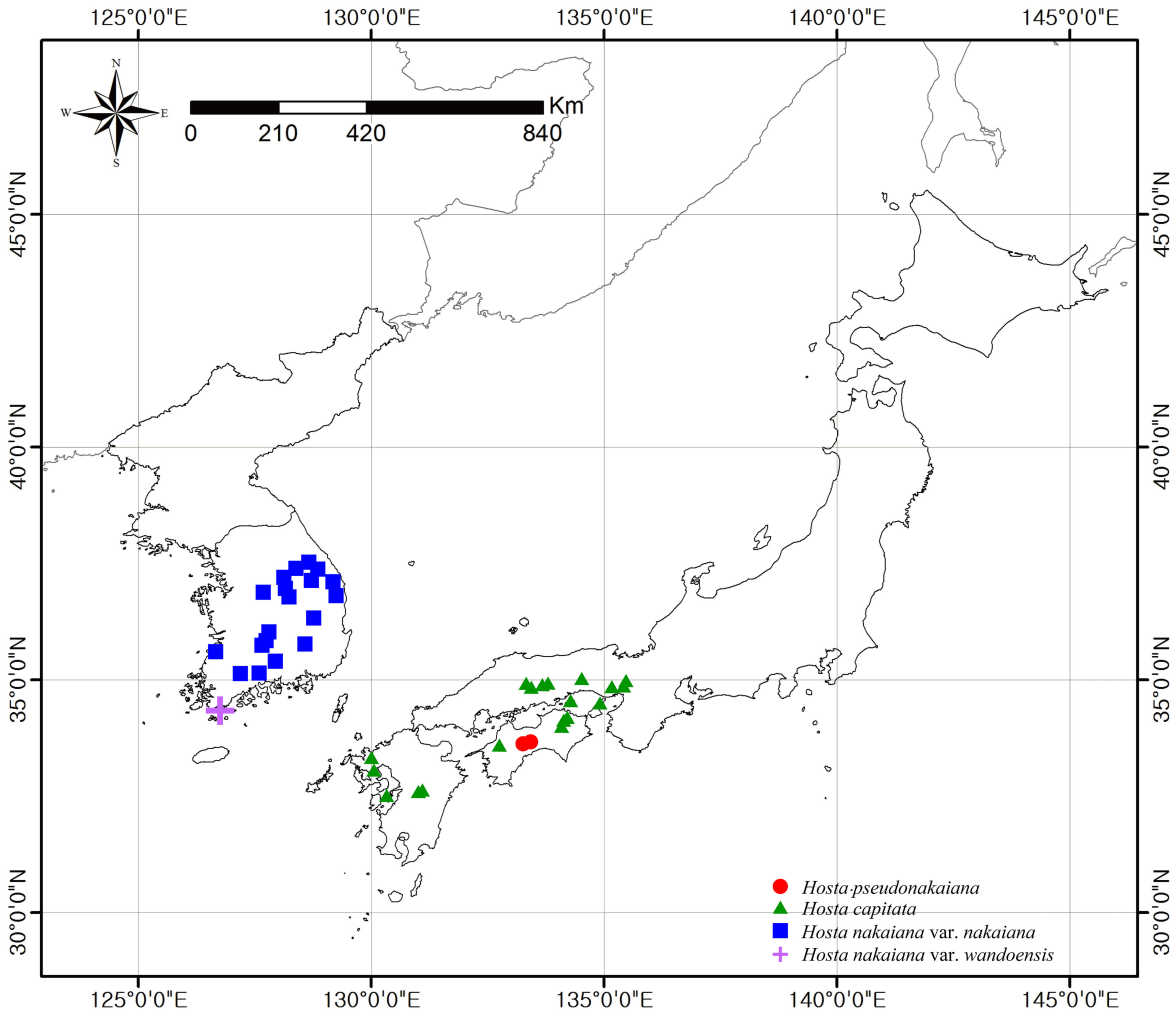
General distribution of *Hosta* sect. *Capitatae* species.

**Figure 9:**
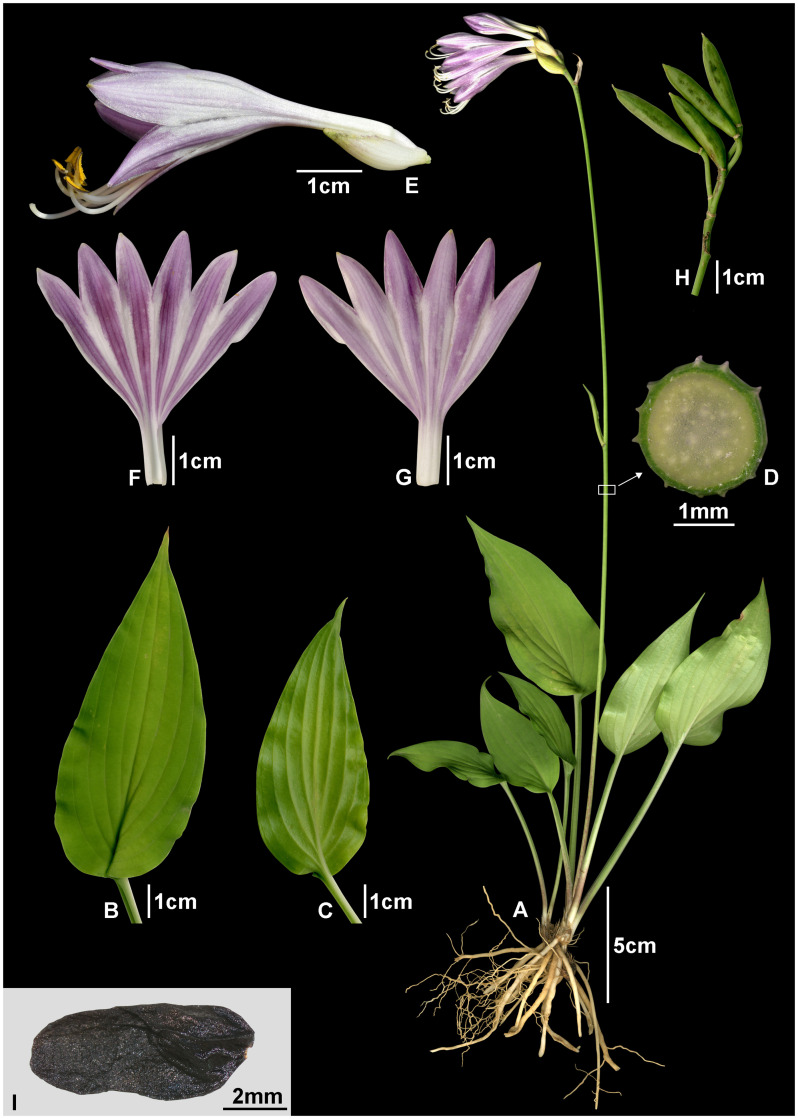
*Hosta pseudonakaiana*. **(A)** habit; **(B, C)** leaf (**B**, adaxial; **C**, abaxial); **(D)** cross-section of scape; **(E)** flower; **(F, G)** perianth (**F**, adaxial; **G**, abaxial); **(H)** capsule; **(I)** seed. Photos from *M.J.Choi & T.Shiga 190706-001*.

*Hosta pseudonakaiana* Shiga & H.J.Choi, sp. nov. (**Figures 7A**, **8**, **9**). — TYPE: JAPAN. Kochi Pref.: Agawa-gun, Niyodogawa-cho, July 6, 2019 [fl], M.J.Choi & T.Shiga 190706-001* (holotype, KB; isotypes, four sheets, KH, KIOM, MBK, NGU)!.

Herbs perennial, rhizomatous. Leaves basal, erect-patent, not shiny; petiole 6.1–20.9 cm long, base purple-dotted; leaf narrowly ovate, green, 9.0–17.0 × 3.3–7.5 cm, veins in 11–15 lateral pairs, base rounded to subcordate, apex acuminate to acute, papillose on abaxial surfaces. Scapes lamellar, ridged longitudinally. Inflorescences compact spike-like raceme, 2–8 flowered; bracts navicular, ovate, acuminate to acute at apex, purplish white, sometimes greenish near apex, deciduous at fruiting. Flowers bell-shaped; perianth pale purple to purple with dark purple nerves, lobes lanceolate; stamens 6, longer than perianth; anthers yellow with purple dots; ovary 3-loculed. Capsules cylindric. Seeds winged, black.

**Figure 10:**
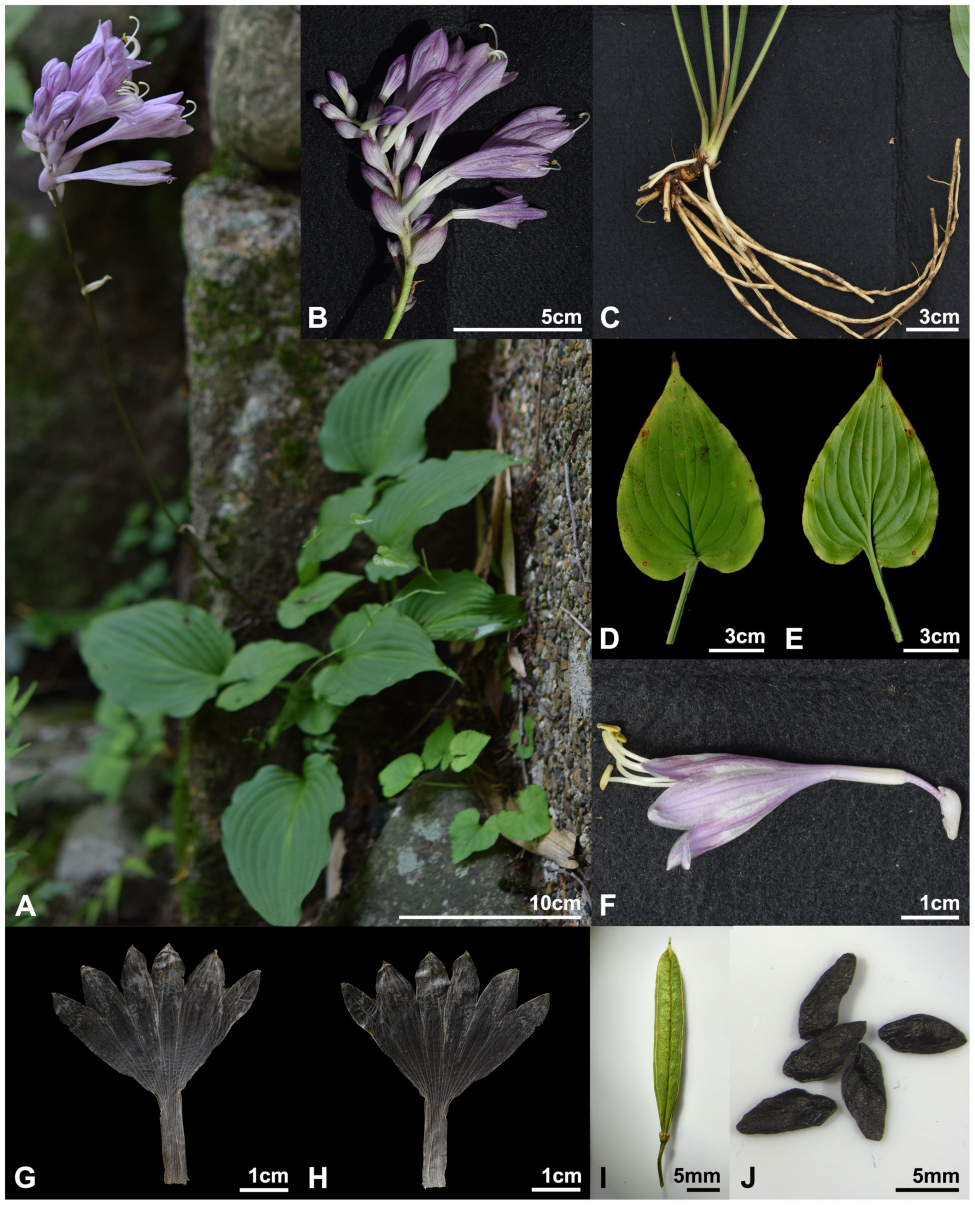
*Hosta capitata*. **(A)** habit; **(B)** inflorescence; **(C)** root; **(D, E)** leaf (**D**, adaxial; **E**, abaxial); **(F)** flower; **(G, H)** perianth (**G**, adaxial; **H**, abaxial); **(I)** capsule; **(J)** seed. Photos from *H.J.Choi 190707-Rokko(Gobe)-001***(A, G, H)** and *H.J.Choi 161008-Iya-001***(B-F, I, J)**.

Note: This new species is morphologically very similar to *H. capitata* in having a ridged scape and compact spike-like racemes; however, several morphological characteristics of the leaves, inflorescences, and perianths differentiate them. In particular, *H. pseudonakaiana* differs in having narrowly ovate leaves vs. ovate to cordate leaves in *H. capitata*, 2–8 flowers per inflorescence vs. 3–13 flowers in *H. capitata*, and lanceolate perianth lobes vs. broadly ovate lobes in *H. capitata* (**Tables 2**, **3**; **Figures 9**, **10**). Schmid (1991) and Sauve et al. (2005) recognized this new species as being taxonomically distinct from *H. capitata*. Morphologically similar specimens were collected in Kagawa and Kumamoto Prefectures, and further analysis using SNP data, similar to the present study, will be necessary in the future to clearly identify these specimens.

Etymology: The specific epithet “*pseudonakaiana*” refers to the fact that this species had been misidentified as *H. nakaiana* in various herbaria.

Local name: Tosano-kanzashi-giboshi

Phenology: Flowering in June to July and fruiting in August to October.

Distribution and habitat: *H. pseudonakaiana* is endemic to Shikoku, Japan (**Figure 8**). This species grows under moist and half-shadowed forest conditions.

Additional specimens examined (Paratypes): JAPAN. Kochi Pref.: Agawa-gun, Ino-cho, Oomorigawa-keikoku (gorge), 7 Jul. 2013 [fl], *Y.Kokami FOS-004908*, *004909* (MBK)!; Nagasawa, 25 Jul. 1983 [fr], *Y.Koukami M83-264* (MBK)!; 04 Jul. 2004 [fl], *T.Nakamura* et al., *FOK-067205* (MBK)!; Choja-ko, 24 Aug. 2016 [fl], *S.Fujii* (NGU)!; 9 Oct. 2016 [fr], *T.Shiga 9261-9264* (NGU)!; 06 Jul. 2019 [fl], *M.J.Choi* et al., 190706-Chojako(Kochi)-001* (CWNU)!; Choja-hei, 6 Jul. 2019 [fl], *T.Shiga 11296* (NGU)!; Takaoka-gun, Niyodo-mura, 9 Aug. 1970 [fr], *N.Fujita & K.Okamura 1* (KYO)!; Gyobuyabu, 9 Sep. 1970 [fr], *N.Fujita & K.Okamura 2* (KYO)!; Shimanto-cho, Eshi, 4 Jul. 1995 [fl], *Y.Koukami 95-130* (MBK)!; Tsuno-cho, Shiraishi-ko, 25 Aug 2016 [fr], S.Fujii, 160825-Takaoka(Kochi)-001* (CWNU)!; Tosa-gun, Motoyama-mura, 26 Jun. 1967 [fl], *T.Yamanaka* (KYO)!.

*Hosta capitata* (Koidz.) Nakai, Bot. Mag. (Tokyo) 44: 514 (Nakai, 1930b). (**Figures 7B**, **8**, **10**). — Basyonym: *H. caerulea* var. *capitata* Koidz., Bot. Mag. (Tokyo) 30: 326 (1916). — TYPE: JAPAN. Tokushima Pref.: Awa-shi, Mima-gun, Higashiiya-mura, June 29, 1915 [fl] (holotype, TI)!.

Herbs perennial, rhizomatous. Leaves basal, erect-patent, not shiny; petiole 14.2–25.9 cm long, base purple-dotted; leaf broadly ovate, green, 9.7–14.9 × 6.5–10.8 cm, veins in 13–19 lateral, base subcordate to cordate, apex acuminate to acute, papillose on abaxial surfaces. Scapes lamellar, ridged longitudinally. Inflorescences compact spike-like raceme, 3–13 flowered; bracts navicular, ovate, acute to obtuse at apex, purplish white, sometimes greenish near apex, deciduous at fruiting. Flowers bell-shaped; perianth pale purple to purple with dark purple nerves, lobes broadly ovate; stamens 6, longer than perianth; anthers yellow with purple dots; ovary 3-loculed. Capsules cylindric. Seeds winged, black.

Note: *H. capitata* was treated as the same species as *H. nakaiana* and *H. pseudonakaiana* in *Hosta* sect. *Capitatae* (Fujita, 1976; Tamura and Fujita, 2016; Jo and Kim, 2017). In previous studies, differences in a few traits, such as the shape of the leaf base and the ratio of leaf blade length to width, were treated as variations within a species. However, according to the MIG-seq results, the genetic distinction was clear within the taxa; therefore, they were treated as separate species (**Figures 4**-**6**). In Japan, the vernacular name “Kanzashi-giboshi” has traditionally been applied to *H. capitata* (e.g., Tamura and Fujita, 2016). This name was originally introduced in association with a description of *H. nakaiana* (Maekawa, 1935). Accordingly, the name “Iya-giboshi” (Koidzumi, 1915), which was historically linked to *H. capitata*, is more appropriate for this species.

Local name: Iya-giboshi (Koidzumi, 1915)

Phenology: Flowering from the end of June to July and fruiting from August to October.

Distribution and habitat: *H. capitata* is endemic to southern Honshu, Shikoku, and Kyushu, Japan (**Figure 8**). This species is typically found in rocky valleys with limestone, granite, serpentine outcrops, and forest margins with open canopies (Fujita, 1976; Tamura and Fujita, 2016).

Additional specimens examined: JAPAN. Hyogo Pref.: Kobe-si, Kita-ku, Kamakura-kyo, 27 Jul. 1977 [fr], *S.Hosomi 17670* (KYO)!; Yamada-cho, Aina, 25 Jun. 1978 [fl], *T.Kobayashi 9381* (KYO)!; 9 Jul. 1978 [fl], N.Fukuoka 9757 (KYO)!; 20 Jul. 2019 [fl], T.Kobayashi 57482 (KYO)!; Dojyo-cho, Shimotanaka, 20 Jun. 1993 [fl], *T.Fujii 3280* (OSA)!; Nada-ku, Higashirokko, Mt. Rokko, 16 Aug. 1992 [fr], *T.Kobayashi 21247* (KYO)!; 2 Jul. 1994 [fl], *T.Kobayashi 25704* (KYO)!; Rokkosan-cho, Mt. Minami-rokko, 04 Sep 2016 [fr], T.Kurazono 160904-Minamirokko-001* (CWNU)!; 07 Jul 2019 [fl], *H.J.Choi* et al., 190707-Rokko(Gobe)-001* (CWNU)!; Mt. Nishitaniyama, 04 Sep 2016 [fr], T.Kurazono 160904-Nishitaniyama-001* (CWNU)!; Takarazuka-si, Takedao, 16 Sep. 1991 [fr], *T.Kobayashi 19596* (KYO)!; Ako-gun, Kamigori-cho, 2 Jul. 1989 [fl], *S.Fujii 2743* (OSA)!; 15 Aug. 1989 [fr], *S.Fujii & M.Kuribayashi 2782* (KYO)!; 11 Oct. 1990 [fr], *H.Nagamasu 4401* (KYO)!; Sep. 1989 [fr], *M.Kuribayashi 230* (KYO)!; 27 Jul. 1991 [fr], *T.Kobayashi 19061* (KYO)!; Oct. 1993 [fr], *T.Takahashi & M.Sawada 3035* (OOM)!; 24 Jun. 1999 [fl], *T.Takahashi 2698* (KYO)!; 29 Jun. 1992 [fl], *N.Fukuoka 13946* (OSA)!; 2 Jul. 1989 [fl], *M.Kuribayashi 204* (OSA)!; 17 Jun. 1995 [fl], *S.Fujii 4280* (OSA)!; border of Kamigori-cho and Mikaduki-cho, Todoma valley, 4 Jun. 2002 [fl], *S.Fujii 9190* (MBK)!; Tsuna-gun, Hokudan-cho, Mt. Joryuji, 30 Jun. 1991 [fl], *T.Kobayashi 18778* (KYO)!; Sanda-shi, Yayoiga-oka, Fukada-park, 29 May 1996 [fl], *T.Fujii 5701* (OSA)!. Okayama Pref.: Mitsu-gun, Kamogawa-cho, Enjyo, 16 Oct. 1971 [fr], *N.Fujita 218* (KYO)!; Kamifusa-gun, 26 Jun. 1989 [fl], *Y.Kohata & S.Kano* (KYO)!; Takahashi-shi, Nariwa-cho, 8 Jul. 1989 [fl], *I.Okubo 327* (KYO)!. Hiroshima Pref.: Hiba-gun, Tojo-cho, 26 Jul. 1987 [fr], *N.Kurosaki 16168* (KYO)!. Tokushima Pref.: Miyoshi-city, Ikeda-cho, Ori, 08 Oct 2016 [fr], *H.J.Choi* et al., 161008-Iya-001* (CWNU)!; Mima-gun, Higashi-Iya-yama-son, 29 Jun. 1915 [fl], *G.Koidzumi* (isotype, KYO)!; Waki-machi, Shimizu, 15 Oct. 1971 [fr], *N.Fujita 258* (KYO)!. Kagawa Pref.: Takamatsu-si, shionoe-cho, Mt. Otaki, 13 Sep. 1961 [fl], *S.Sakaguchi* (KYO)!. Ehime Pref.: Kita-gun, Uchiko-cho, 19 Jul. 1973 [fl], *T.Yamanaka 64945* (KYO)!. Saga Pref.: Imari-si, Ohkawauchi-cho, 6 Sep. 2021 [fr], *Y.Inoue SS210906* (KYO)!; Fujitsu-gun, Tara-cho, Mt. Tara, 1 Nov. 1919 [fr], *I.Kawauchi* (KYO)!; 13 Oct. 1992 [fr], *Z.Tashiro* (KYO)!; 22 Jul. 2011 [fr], *Y.Inoue* (KYO)!. Kumamoto Pref.: Yatsushiro-si, Izumi-mura, 10 Aug. 1977 [fl], *H.Tomita 1295* (KYO)!. Miyazaki Pref.: Higashiusuki-gun, Shiiba-mura, Kiritachi-goe, Mt. Shiroiwa, 15 Aug. 1961 [fr], *M.Hotta 6475* (KYO)!; 24 Sep. 1988 [fr], *M.Furuse 54560* (KYO)!.

*Hosta nakaiana* F.Maek., J. Jap. Bot. 11(10): 687. (1935) (**Figure 7C**). — TYPE: KOREA. Jeollanam-do: Mt. Baegunsan, July 6, 1935 [fl] ((holotype, TI)!).

A key to varieties of *Hosta nakaiana*

1. Inflorescence compactly spike-like, scape lamellar ridged, 2.6 (6.4 ± 2.0) 13.9 cm long, 2 (7.3 ± 2.3) 13 flowers per inflorescence, flowering at the end of June to July ——————— var. *nakaiana*

1. Inflorescence loosely spike-like, scape smooth, 3.6 (10.2 ± 3.0)16.9 cm long, 7 (10.3 ± 1.8) 14 flowers per inflorescence, flowering at early to end of June ———————————— var. *wandoensis*

**Figure 11:**
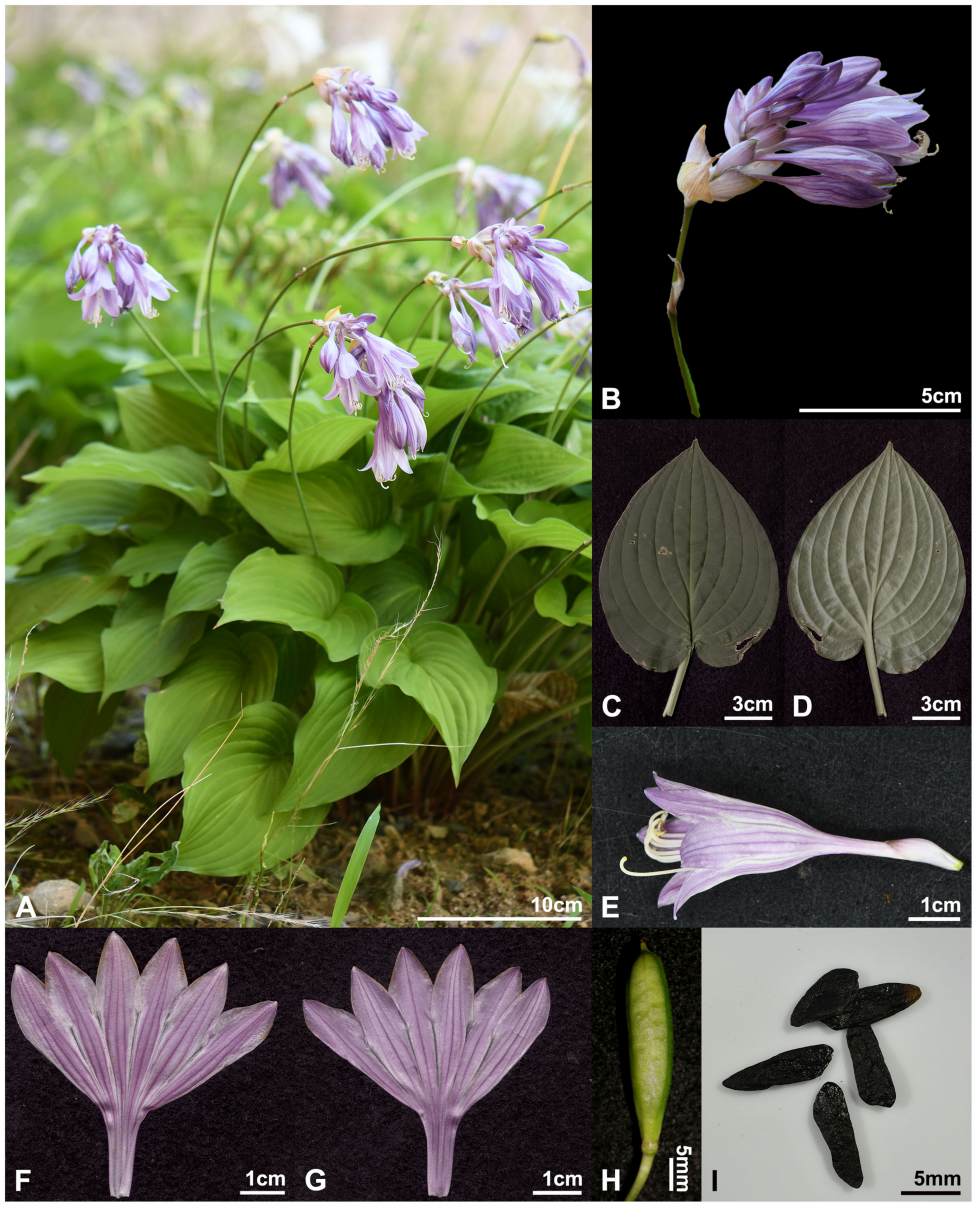
*Hosta nakaiana* var. *nakaiana*. **(A)** habit; **(B)** inflorescence; **(C, D)** leaf (**C**, adaxial; **D**, abaxial); **(E)** flower; **(F, G)** perianth (**F**, adaxial; **G**, abaxial); **(H)** capsule; **(I)** seed. Photos from *H.J.Choi 150806-Gwangyangsi(Baegunsan)-046*.

*Hosta nakaiana* var. *nakaiana* (**Figures 8**, **11**).

Herbs perennial, rhizomatous. Leaves basal, erect-patent, not shiny; petiole 6.2–36.9 cm long, base purple-dotted; leaf broadly ovate, green, 7.7–16.9 × 5.2–14.5 cm, veins in 13–21 lateral, base subcordate to cordate, apex acuminate to acute, papillose on abaxial surfaces. Scapes lamellar, ridged longitudinally. Inflorescences compact spike-like raceme, 2–13 flowered; bracts navicular, ovate, acute to obtuse at apex, purplish white, sometimes greenish near apex, deciduous at fruiting. Flowers bell-shaped; perianth pale purple to purple with dark purple nerves, lobes broadly ovate; stamens 6, longer than perianth; anthers yellow with purple dots; ovary 3-loculed. Capsules cylindric. Seeds winged, black.

Note: Despite their morphological similarities (**Tables 2**, **3**, **Figure 2**), *H. nakaiana* and *H. capitata* were genetically distinct groups based on the results of the MIG-seq analyses (**Figures 4**-**6**). In addition, *H. nakaiana* and *H. capitata* are distributed in Korea and Japan, respectively.

Local name: Ilwol-bibichu

Phenology: Flowering from the end of June to July and fruiting from August to October.

Distribution and habitat: *H. nakaiana* var. *nakaiana* is endemic to Korea (**Figure 8**). This species grows in rocky valleys, mountain summits, ridges, and slopes (Jo and Kim, 2017).

Additional specimens examined: KOREA. Gangwon-do: Yeongwol-gun, Sangdong-eup, Taebaeksan-ro, 3432-74, 37°04’25.3”N, 128°49’53.3”E, 1,174 m a.s.l., 27 Jul. 2008 [fl], J.O.Hyun, 1001019* (KH)!; 37°04’02.4”N, 128°50’00.5”E, 1,263 m a.s.l., 27 Jul. 2008 [fl], J.O.Hyun, 1001023* (KH)!; Yeongwol-eup, Munsan-ri, San161, 37°16’34.9”N, 128°29’58.5”E, 769 m a.s.l., 17 Jul. 2008 [fl], J.O.Hyun & H.J.Gwon, NAPI-0414* (KH)!; Jeongseon-gun, Yeoryang-myeon, Bongjeong-ri, San1, 37°26’34.7”N, 128°45’30.4”E, 1,053 m a.s.l., 30 Jul. 2010 [fl], K.S.Kim & H.J.Kim, K0801020* (KH)!; Jeongseon-eup, 26 Jun. 2013 [fl], *B.U.Oh*, KH00719* (KH)!; Taebaek-si, Changjuk-dong, san76, 37.23967°N, 128.91963°E, 1,161 m a.s.l., 29 Jul. 2016 [fl], *H.J.Choi* et al., 160729-Taebaeksi(Daedeoksan)-001* (CWNU)!. Chungcheongbuk-do: Jecheon-si, Cheongpung-myeon, Hakhyeon-ri, 289-1, 37°00’40”N, 128°12’56”E, 279 m a.s.l., 14 Aug. 2006 [fr], *G.H.Nam & J.E.Koh*, CHJ60438* (KH)!; San6-1, 37°01’36.4”N, 128°13’22.8”E, 780 m a.s.l., 18 Oct. 2006 [fr], *S.H.Park* et al., ParkSH63602* (KH)!; Goesan-gun, Chilseong-myeon, Saeun-ri, San5-1, 36°44’06.8”N, 127°53’11.7”E, 553 m a.s.l., 21 Jun. 2006, *W.K.Paik* et al., Sangjusi (Songnisan)-060621-112* (KH)!; Goesan-gun, Mt. Songnisan, 26 Jun. 2013 [fl], *B.U.Oh* et al., Goesangun-130627-133* (KH)!; 5 Aug. 2013 [fr], *B.U.Oh* et al., Goesangun-130805-008* (KH)!; Boeun-gun, Mt. Songnisan, 11 Oct. 2002 [fr], *Y.M.Kim* et al., L-60077* (KH)!; 23 Jun. 2013 [fl], *B.U.Oh* et al., Boeungun-130623-012* (KH)!. Jeollabuk-do: Muju-Gun, Mupung-myeon, Samgeo-ri, San1-11, 35°52’52.3”N, 127°50’26.9”E, 938 m a.s.l., 23 Jun. 2008 [fl], *J.O.Hyun* et al., NAPI-0293* (KH)!; Jangsu-gun, Gyebuk-myeon, Yangak-ri, San35-2, 35°46.563’N, 127°41.061’E, 1,252 m a.s.l., 07 Aug. 2013 [fr], *H.J.Choi* et al., 130807_hjchoi065* (KH)!; Wanju-gun, Mt. Moaksan, Bukbong-Moakjeong, 30 Jun. 2013 [fl], *B.U.Oh*, 130630-Moaksan-027* (KH)!; 30 Jun. 2013 [fl], *B.U.Oh*, 130630-Moaksan-023* (KH)!; Gochang-gun, Asan-myeon, Samin-ri, San35, 35.51770°N, 126.57699°E, 423 m a.s.l., 28 Sep. 2013 [fr], *H.J.Choi* et al., 130928-Gochanggun(Seonunsan)-020* (CWNU)!. Jeollanam-do: Suncheon-si, Juam-myeon, Ullyong-ri, San142. 35.116342°N, 127.204698°E, 193 m a.s.l., 24 Jun. 2009 [fl], *Y.H.Cho* et al., WR-090624-159* (KH)!; Gwangyang-si, Jinsang-myeon, Beakhak-ro, 1243, 35°06’22”N, 127°37’19”E, 1,205 m a.s.l., 12 Aug. 2010 [fr], *D.O.Lim* et al., LIM1462* (KH)!; Eochi-ri, San308, 35.10805°N, 127.62758°E, 1,084 m a.s.l., 06 Aug. 2015 [fr], *H.J.Choi* et al., 150806-Gwanyangsi(Baegunsan)-046* (CWNU)!. Gyeongsangbuk-do: Yecheon-gun, Yongmun-myeon, Nosa-ri, 290. 36°40’55.15”N, 128°21’41.11”E, 236 m a.s.l., 06 Jul. 2008 [fl], *C.G.Jang* et al., VP-KNU-368061-0011* (KH)!; Uiseong-gun, Gaeum-myeon, Hyeolli-ri, 11-6, 36°12’12.6”N, 128°47’45.5”E, 879 m a.s.l., 14 Jul. 2006 [fl], *C.G.Jang*, Jang310* (KH)!; Yeongyang-gun, Subi-myeon, Suha-ri, San26-1, 36°49’03.4”N, 129°16’08.9”E, 396 m a.s.l., 31 Aug. 2013 [fr], *K.H.Bae* et al., KH-130148* (KH)!; Bonghwa-gun, Chunyang-myeon, Seobyeok-ri, San103, 37°01’16.8”N, 128°47’12.5”E, 992 m a.s.l., 19 Jul. 2010 [fl], *S.H.Oh* et al., C100836* (KH)!; Seokpo-myeon, Banya-gil, 176-37, 37°02’15.1”N, 129°09’77.9”E, 883 m a.s.l., 20 May 2010 [fl], *K.H.Bae* et al., KH-1000181* (KH)!; Mungyeong-si, Dongno-myeon, Saengdal-ri, 493, 36°48’58.4”N, 128°15’40.6”E, 761 m a.s.l., 14 Jul. 2006 [fl], *W.K.Paik* et al., Mungyeongsi (Hwangjangsan)-060714-808* (KH)!; Seongju-gun, Geumsu-myeon, Muhak-ri, Mt. Baebawisan, 13 Oct. 2007 [fr], *B.U.Oh*, 071013-Baebawisan-009* (KH)!; Gimcheon-si, Daedeok-myeon, Deoksan-ri, San42-4, 35.92462°N, 127.90825°E, 495 m a.s.l., 29 Jun 2013, *H.J.Choi* et al., 130629-Gimcheonsi(Daedeoksan)-019* (CWNU)!; Daehang-myeon, Jurye-ri, San1, 36.10550°N, 127.96708°E, 923 m a.s.l., 02 Jul 2012 [fl], *H.J.Choi* et al., 120702-Gimcheonsi(Hwangaksan)-543* (CWNU)!; Daegu-si, Dalseong-gun, Gachang-myeon, Samsan-ri, San171-1, 35.72109°N, 128.68133°E, 452 m a.s.l., 28 Jun 2013 [fl], *H.J.Choi* et al., 130628-Daegusi(Sangwonsan)-079* (CWNU)!. Gyeongsangnam-do: Sancheong-gun, Samjang-myeon, Honggye-ri, San336, 35°20’44.97”N, 127°51’24.31”E, 396 m a.s.l., 20 Jun. 2007 [fl], *J.C.Yang*, YangJC 70056* (KH)!; Danseong-myeon, Un-ri, San147, 35°20’51.51”N, 127°52’55.14”E, 687 m a.s.l., 27 Jun. 2007 [fl], *J.C.Yang*, YangJC 70199* (KH)!; San1, 35°20’41.03”N, 127°53’34.81”E, 360 m a.s.l., 27 Jun. 2007 [fl], *J.C.Yang*, YangJC 70206* (KH)!; Sangcheong-eup, Nae-ri, San204, 35°22’48”N, 127°52’37”E, 200 m a.s.l., 20 Jun. 2007 [fl], *J.C.Yang*, YangJC 70011* (KH)!; San158-12, 35°23’00.83”N, 127°52’16.13”E, 350 m a.s.l., 28 Jun. 2007 [fl], *J.C.Yang*, YangJC 70274* (KH)!; Geochang-gun, Goje-myeon, Bongsan-ri, San252, 35°52’29.0”N, 127°51’20.9”E, 963m a.s.l., 24 Jun. 2008 [fl], *J.O.Hyun* et al., NAPI-0304* (KH)!.

**Figure 12:**
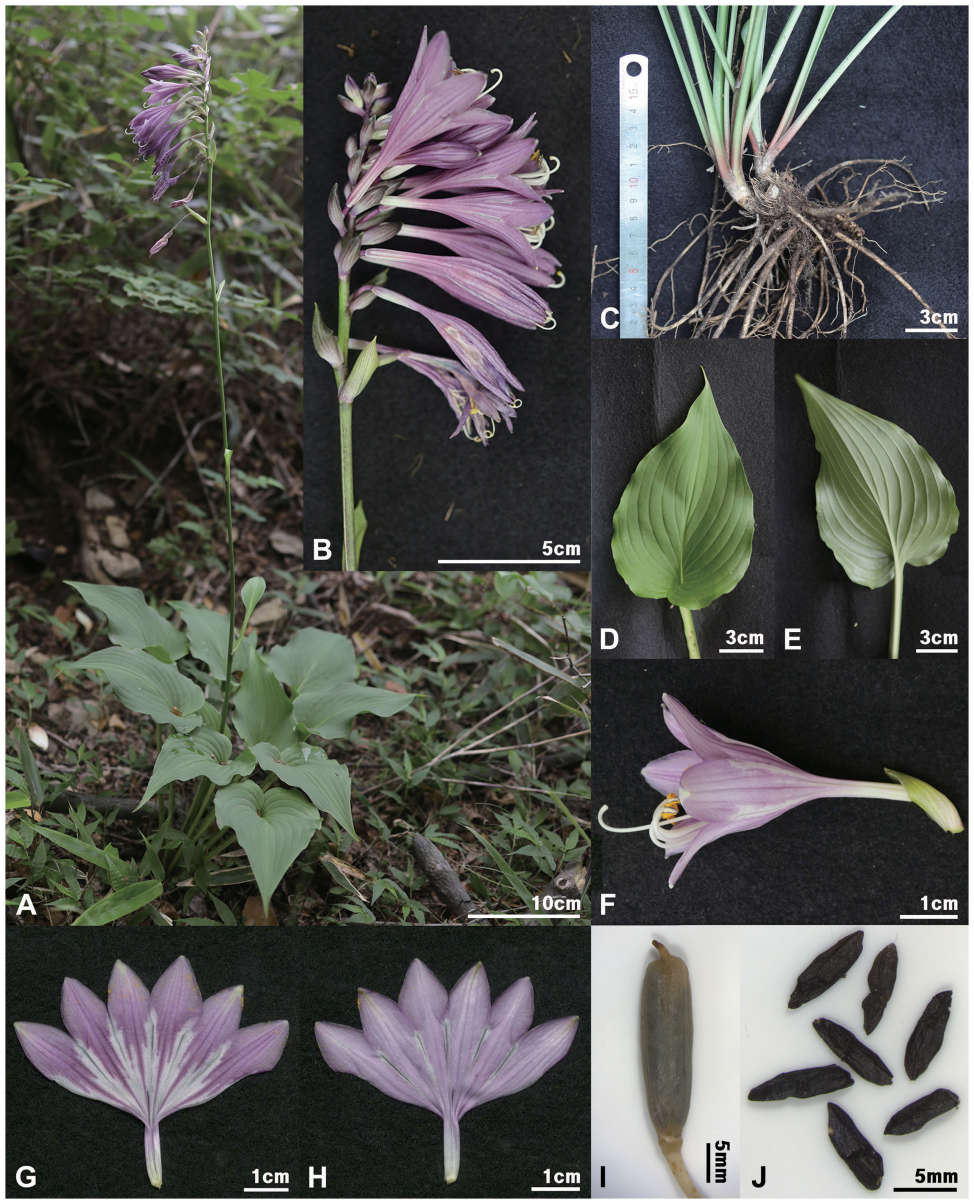
*Hosta nakaiana* var. *wandoensis*. **(A)** habit; **(B)** inflorescence; **(C)** root; **(D, E)** leaf (**D**, adaxial; **E**, abaxial); **(F)** flower; **(G, H)** perianth (**G**, adaxial; **H**, abaxial); **(I)** capsule; **(J)** seed. Photos from *H.J.Choi Wando-210623-001*.

*Hosta nakaiana* var. *wandoensis* H.J.Choi, var. nov. (**Figures 7D**, **8**, **12**). — TYPE: KOREA. Jeollanam-do: Wando-gun, Wando-eup, Daesin-ri, San8-1, 34.34675°N, 126.69531°E, 516 m a.s.l., June 23, 2021 [fl], Wando-210623-001* (holotype, HIBR; isotypes, three sheets, KB, KH, KIOM)!.

Herbs perennial, rhizomatous. Leaves basal, erect-patent, not shiny; petiole 10.1–27.9 cm long, base purple-dotted; leaf broadly ovate, green, 9.7–19.5 × 6.1–12.2 cm, veins in 15–23 lateral, base subcordate to cordate, apex acuminate to acute, papillose on abaxial surfaces. Scapes lamellar ridged. Inflorescences loose, spike-like racemes, 7–14 flowered; bracts navicular, ovate, acute to obtuse at apex, purplish-white, sometimes greenish near apex, deciduous at fruiting. Flowers bell-shaped; perianth pale purple to purple with dark purple nerves, lobes broadly ovate; stamens 6, longer than perianth; anthers yellow with purple dots; ovary 3-loculed. Capsules cylindric. Seeds winged, black.

Note: This new variety is morphologically very similar to *H. nakaiana* var. *nakaiana* in having a ridged scape and bell-shaped flowers, but several morphological characteristics of the shapes of leaves and inflorescences easily differentiate them (**Figures 7**, **11**, **12**): var. *wandoensis* differs by having broadly ovate leaves and cordate base vs. ovate to cordate leaves and subcordate to cordate base in var. *nakaiana*. In addition, flowering from early to the end of June vs. flowering from the end of June to July in var. *nakaiana* and 7–14 flowers per inflorescence with a loose spike-like raceme vs. 2–13 flowers per inflorescence with a compact spike-like raceme in var. *nakaiana* (**Tables 2**, **3**).

Etymology: The specific epithet was derived from the name of the type locality (Wando Island).

Local name: Wando-bibichu

Phenology: Flowering in June and fruiting in July to August.

Distribution and habitat: *H. nakaiana* var. *wandoensis* is endemic to Korea (Wando, Jeollanam-do; **Figure 8**). This species grows in shaded mountain ranges and open habitats on mountain summits.

Additional specimens examined (Paratypes): KOREA. Jeollanam-do: Wando-gun, Wando-eup, Daesin-ri, San8-1, 34.34675°N, 126.69531°E, 516 m a.s.l., 19 Jun 2019 [fl], *H.J.Choi* et al., ﻿190619-Wandogun(Sangwangbong)-001* (CWNU)!*; 20 Jun 2025 [fl], *H.J.Choi* et al., ﻿250620-Wandogun(Sangwangbong)-001 (CWNU)!.

## Data Availability

The datasets presented in this study can be found in online repositories. The names of the repository/repositories and accession number(s) can be found below: https://www.ncbi.nlm.nih.gov/, PRJNA1284551.
