# Supplementary material for: Delimiting species boundaries in Hosta section Capitatae (Asparagaceae) using MIG-seq and morphological analyses: taxonomic revision with new taxa from Korea and Japan
Source: Front Plant Sci. 2026 Jan 21;16:1668561. doi: 10.3389/fpls.2025.1668561 (PMC12868265; doi:10.3389/fpls.2025.1668561)
Supplement: Supplementary file 1 [file DataSheet1.pdf]

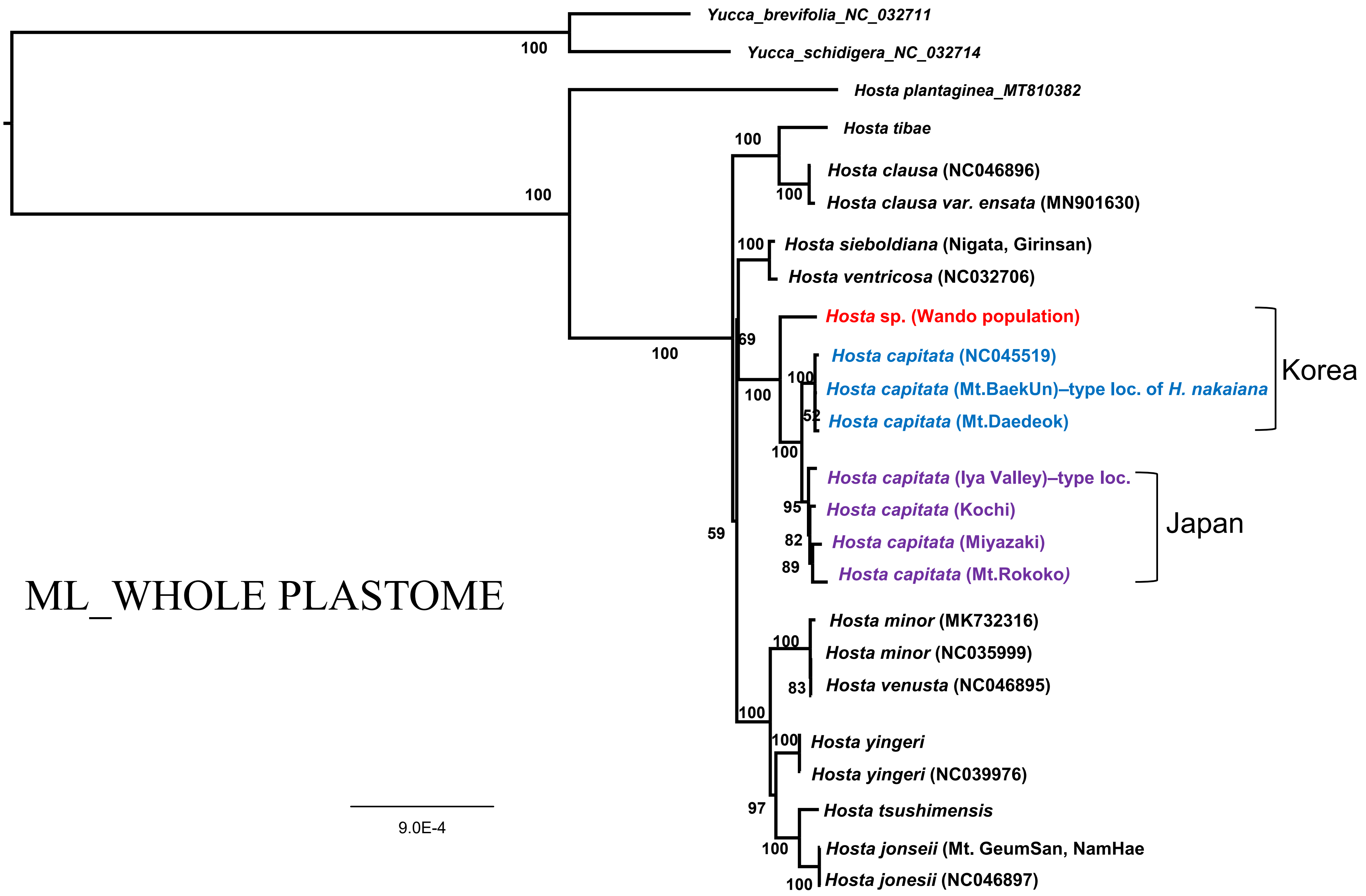

Supplementary Figure 1. Maximum likelihood tree inferred with 22 *Hosta* populations and two *Yucca* populations using IQ-TREE v.1.4.2. The numbers on the node represent bootstrap support values from 1,000 bootstrap replicates.
